# Cleavable Additives for Deconstructable, Recyclable Polyurethane Thermosets

**DOI:** 10.1021/acscentsci.5c00689

**Published:** 2025-07-23

**Authors:** Kwangwook Ko, David J. Lundberg, Valerie L. Lensch, Yasmeen S. AlFaraj, Keith E. L. Husted, Jacob P. Brutman, Alaaeddin Alsbaiee, Patrick N. Hamilton, Suong T. Nguyen, Jeremiah A. Johnson

**Affiliations:** † Department of Chemistry, 2167 Massachusetts Institute of Technology, 77 Massachusetts Avenue, Cambridge, Massachusetts 02139, United States; ‡ Department of Chemical Engineering, Massachusetts Institute of Technology, 25 Ames Street, Cambridge, Massachusetts 02139, United States; § BASF Corporation, 1609 Biddle Avenue, Wyandotte, Michigan 48192, United States

## Abstract

Polyurethane (PU) thermosets, particularly those derived from aliphatic components, are challenging to chemically deconstruct due to their permanent cross-linking. Current approaches to impart deconstructability typically rely on complete substitution of network precursors with cleavable analogs, limiting practicality. Cleavable additives (CAs) offer a potentially simple and cost-effective alternative, yet their application has been largely confined to chain-growth networks and remains unexplored in end-linked systems such as PUs. Here, we present a generalizable reverse gel-point theory that predicts the minimum CA loading required for deconstruction of end-linked networks. We validate this framework experimentally through the incorporation of two classes of silyl ether-based CAs—bifunctional cleavable strands (BCSs) and trifunctional cleavable junctions (TCJs)—into PU thermosets. Both additives enable selective PU dissolution at low loadings (5–12 wt %), with TCJs demonstrating enhanced efficiency. The combined use of BCSs and TCJs also allows fine-tuning of material properties. Furthermore, we show that polyol fragments generated from the deconstruction of TCJ-containing PUs can be chemically repolymerized to regenerate PU materials without loss of mechanical performance over multiple cycles. This work establishes CAs as a viable strategy for advancing PU circularity and offers a foundational framework for their broader application in end-linked polymer networks.

## Graphical abstract

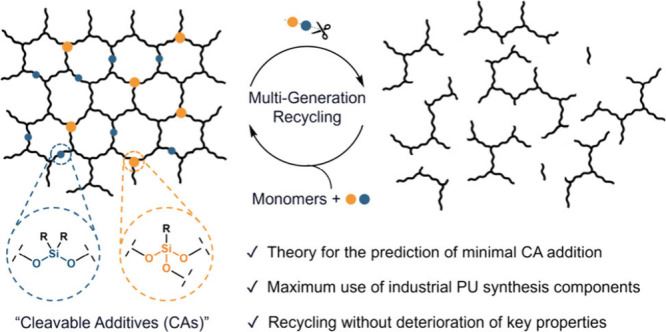

## Introduction

Millions of tons of polyurethanes (PUs) are produced every year for use as adhesives, coatings, and foams, making them the sixth-most produced polymers today.^1–4^ Their commercial success is due to their desirable thermomechanical properties, facile synthesis, and thermal stability.^5,6^ As is common for many synthetic polymers, however, these beneficial features have a key drawback: an ever-growing volume of nondegradable PU waste ends up in landfills or waterways.^7^ For instance, more than 1 million tons of PU waste is deposited into landfills annually in the United States alone.^8,9^ More than 96% of the PU market is related to thermosets (i.e., permanently cross-linked polymer networks), which cannot undergo conventional thermomechanical recycling due to their inherent resistance to melting or dissolution into reprocessable forms.^10^ Thus, strategies for imparting circularity to PU thermosets could contribute substantially to a circular plastics economy.

PUs differ from many other commodity plastics in that their formulations cover a wide range of compositions and properties, rendering a general approach to their circularity difficult.^5,11^ For example, the criteria for reusing insoluble and rigid PU insulation may differ greatly from those required to recycle soluble adhesives, even if both were synthesized from chemically similar constituents.^4^ While pyrolysis is a promising strategy to convert some forms of plastic waste into building blocks for fuels or other chemicals, such procedures are often energy intensive for PUs (requiring temperatures greater than 800 °C) and yield low-value products (e.g., char).^12^ Chemical deconstruction of PUs by cleavage of their urethane bonds via hydrolysis, acidolysis, or aminolysis is possible;^13–16^ however, these processes typically require harsh conditions (e.g., strong acids) and high temperatures, and the recovered products are of limited purity.^17,18^ Although recent advances in catalysis have shown promise for the depolymerization of PUs at milder temperatures, successful deconstruction and recovery of products suitable for recycling has largely been restricted to PUs synthesized from aromatic isocyanates and has not yet proven broadly applicable to those derived from aliphatic counterparts.^19–21^ PUs constructed from polyols containing orthogonally cleavable bonds can overcome this challenge,^22–25^ enabling mild deconstruction to recover building blocks for new PU synthesis, but so far, such approaches have involved the use of bespoke cleavable polyols as the sole cleavable components for PU synthesis, precluding the use of common industrially employed polyols (Figure 1A). By contrast, thermal and/or catalytic *exchange* of urethane bonds can be leveraged for mechanical recycling of certain PU types but may not be applicable to all classes and may not facilitate chemical recycling.^26–32^ Thus, general and efficient strategies for chemical recycling of PUs composed primarily of industrially relevant monomers are desirable.

**Figure 1:**
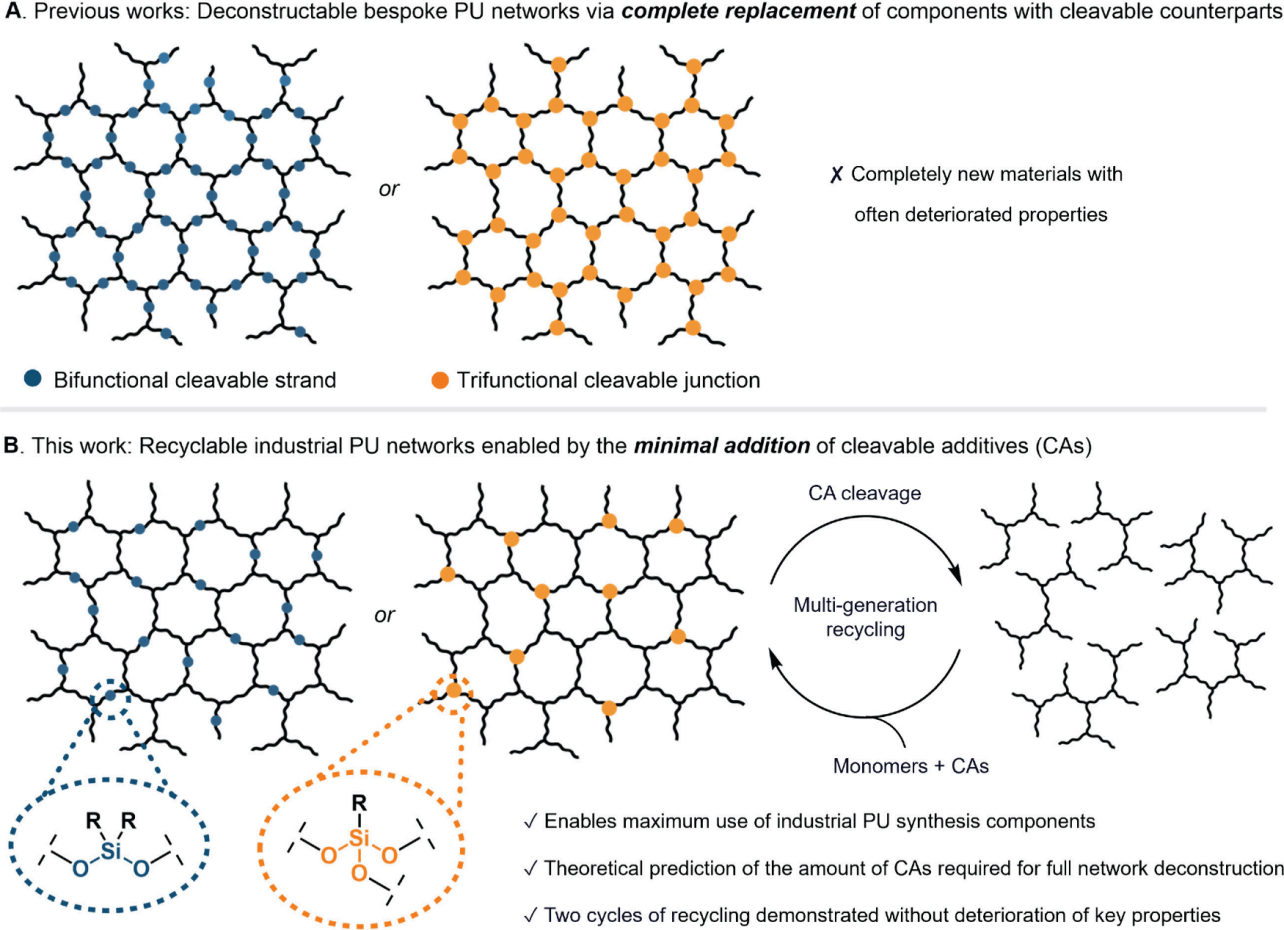
Contextualization of this study: (A) Previous works on deconstructable PU networks relied on the wholesale replacement of network strands or junctions with cleavable variants, which precluded the use of common PU building blocks. (B) This work introduces BCS and TCJ as “cleavable additives” (CAs) that incorporate cleavable linkages into otherwise nondeconstructable PUs, thereby enabling the chemical recycling of PUs manufactured primarily from common components.

We have put forth the concept of “drop-in” cleavable additives (CAs) for manufacturing polymers with triggered deconstructability and new options for chemical circularity.^33–40^ By leveraging CAs, it is possible to produce materials with similar compositions and properties to those used today, but with the option for selective dissolution at end-of-life to yield oligomeric fragments that can be repolymerized to generate recycled materials with properties similar to the virgin materials. Optimal CAs should be cheap to produce, appropriately reactive under existing manufacturing protocols, not detrimental to the desirable properties of the polymers to which they are added, and able to impart selective cleavability to produce well-defined products for subsequent chemical recycling. Over the past several years, we have developed CAs for various classes of thermosets and thermoplastics produced using *chain-growth* polymerizations (e.g., radical polymerization and ring-opening metathesis polymerization), where reactivity ratios and cross-link densities ultimately dictate the amounts of CA needed to achieve full deconstruction (e.g., dissolution), driving the search for CAs with optimal copolymerization reactivity.^36,38,41^ By contrast, the impacts of CAs in the context of *end-linked* polymer network topologies, which encompass PU thermosets, have not been fully investigated, suggesting a rich new area for novel CA design.

Here, we study how CAs impact the deconstructability, properties, and chemical recyclability of PU thermosets composed primarily of traditional noncleavable polyols and diisocyanates. Specifically, we introduce two different strategies for CA design: a “bifunctional cleavable strand” (BCS) approach and a “trifunctional cleavable junction” (TCJ) approach (Figure 1B), both utilizing Si–O bonds as the cleavable linkages due to their stability under typical use conditions yet capability to undergo selective, efficient cleavage reactions when desired.^25,33,42–45^ BCSs are linear diols that contain cleavable Si–O bonds, leading to strand scission in PU networks. TCJs are branched triols with cleavable Si–O cores, resulting in junction scission–i.e., cleavage at the intersection of three strands–in PU networks. We hypothesized that these differences in the locations of cleavable bonds would lead to pronounced impacts on the efficacy of PU deconstruction and chemical recycling, and could provide a strategy for tuning PU material properties.

To elucidate the differences between BCS and TCJ CAs in imparting deconstructability, we develop a reverse gel-point theory model for end-linked networks that predicts the minimum CA loading required for network deconstruction at a specified cross-linker level. We validate this theoretical framework by synthesizing PUs from commercially available polyols and diisocyanates, incorporating either or both BCS and TCJ CAs, and assessing their deconstructability. The resulting PUs, incorporating low levels of CAs (5–12 wt %), undergo mild, Lewis acid-mediated methanolysis to yield well-defined, soluble polyol fragments. Notably, TCJs exhibit enhanced efficiency over BCSs, achieving deconstructability at lower additive loadings. We also find that these CAs exert opposing effects on PU mechanical properties, enabling both the preservation of virgin PU characteristics when used together and tunability when desired. Using TCJ-based networks, we demonstrate that PU thermosets can be chemically recycled over multiple cycles without deterioration of key properties across successive generations. Overall, this work introduces CAs that facilitate the synthesis of chemically recyclable PUs using low-cost components and established workflows, while offering fundamental insights into CA design for end-linked networks with potential applicability to other material classes such as epoxies and thiol–ene networks.

## Results and Discussion

### Reverse Gel-Point Model for the Deconstruction of PU Thermosets

To evaluate the feasibility of using CAs for the deconstruction of PU networks, we developed a reverse gel-point model based on the Miller-Macosko theory to predict the minimum CA loading required for complete deconstruction (i.e., dropping below the gel point).^46,47^ Assuming full conversion of functional groups and equal alcohol and isocyanate stoichiometries, the model predicts that network dissolution occurs when the condition in eq 1 is met:

1 $$\left(f_{e}-1\right)\left(g_{e}-1\right)\leq 1$$

where *f*_*e*_ and *g*_*e*_ represent the weight-average branching functionalities of the alcohol and isocyanate components, respectively. In a PU network formed using diisocyanates, diols, and triols as cross-linkers, *g*_*e*_ = 2. Therefore, the reverse gel-point, where full deconstruction is achieved, is reached when *f*_*e*_ is less than or equal to 2 (see SI for further discussion).^33^ Moreover, when a portion of the diol and triol is substituted with CAs (BCSs and TCJs, respectively), eq 1 can be rearranged to derive eq 2, which predicts the reverse gel point (see SI for a detailed derivation):

2 $$2X_{BCS}+6X_{TCJ}\geq 3X_{cross‐link}$$

where *X*_*BCS*_, *X*_*TCJ*_, and *X*_*cross*‑*link*_ represent the molar amounts of BCS, TCJ, and the total cross-linking component (i.e., triols and TCJ) used in PU synthesis. This equation provides a general criterion for determining the required amounts of BCS and TCJ CAs needed to achieve network deconstruction based on cross-linker loading. When BCSs are used exclusively as the CAs, eq 2 simplifies to eq 3.

3 $$X_{BCS}\geq \frac{3}{2}X_{cross‐link}$$

Thus, deconstructability is predicted to occur when the amount of BCS added is equal to 1.5 times the amount of triols (i.e., nondegradable cross-linkers). Conversely, when TCJs are used exclusively as the CAs, eq 2 reduces to eq 4:

4 $$X_{TCJ}\geq \frac{1}{2}X_{cross‐link}$$

Here, the cross-linking component consists of TCJ and nondegradable cross-linkers. Therefore, network deconstruction is expected when half of the cross-linking components are cleavable (i.e., TCJ). In other words, the model predicts that one-third the amount of TCJ, compared to BCS, is sufficient to achieve full dissolution of PU materials with the same cross-link density. Overall, the reverse gel-point model highlights the potential of CAs to enable complete network deconstruction without requiring wholesale replacement of network components.

### Design and Synthesis of CAs

We aimed to develop CAs that can be synthesized from cost-effective starting materials, offer robust stability under conditions typically encountered in PU applications, and undergo selective and efficient cleavage under mild chemical conditions. Silyl ethers were selected as the cleavable functionalities due to their facile synthesis, high bond strengths, and thermal stabilities, as well as the availability of numerous well-established mild Si–O bond cleavage conditions.^48–52^ Accordingly, we synthesized a BCS, **Et**_**2**_ **Si­(OC**_**6**_ **H**_**12**_ **OH)**_**2**_, via a one-step reaction between hexanediol and dichlorodiethylsilane, achieving a 52% yield (Figure 2A). Notably, hexanediol is nontoxic and is widely used in PU production.^53^

**Figure 2:**
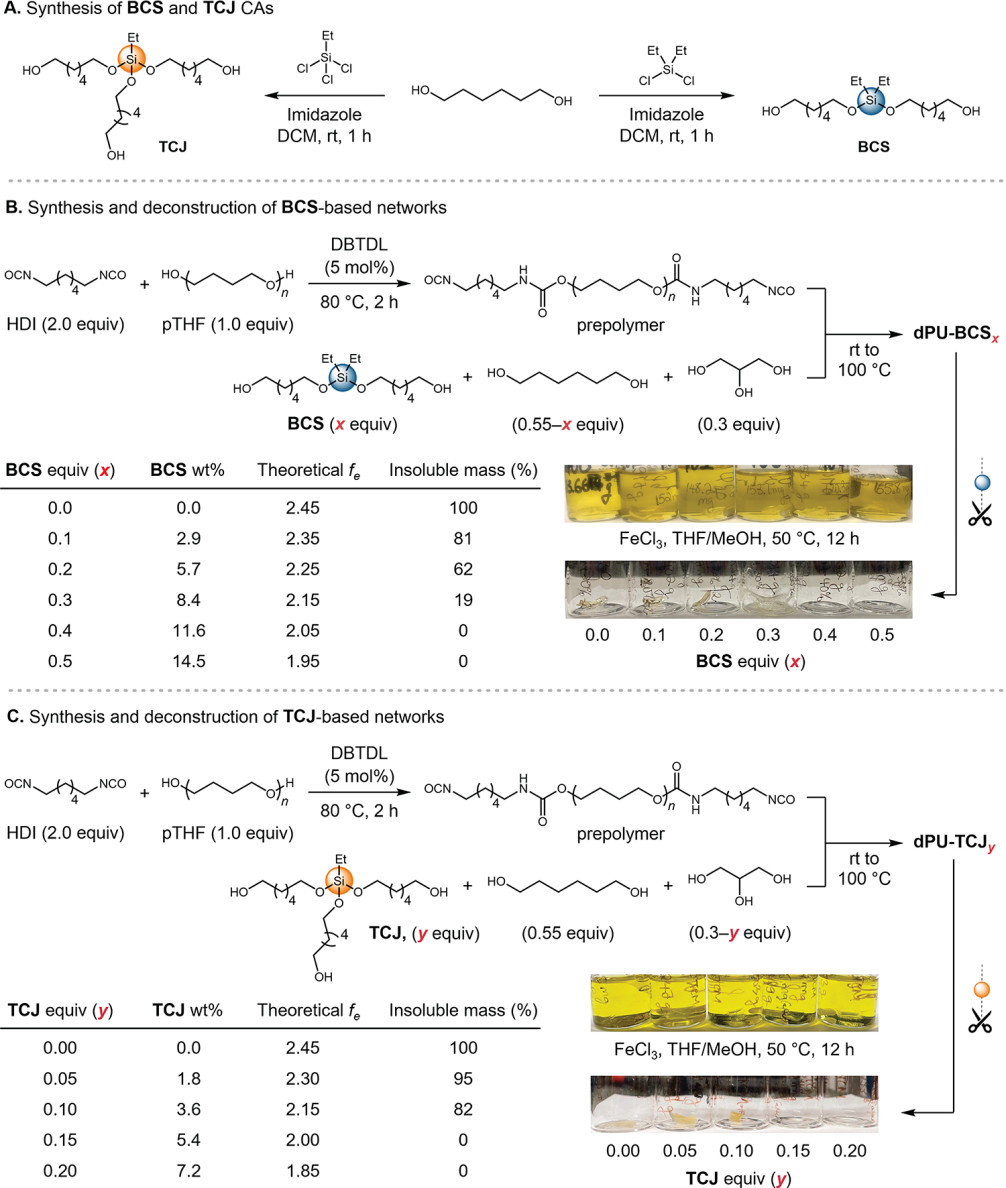
Synthesis and deconstruction of BCS- and TCJ-based PU networks. (A) BCS and TCJ CAs were each synthesized in a single step from readily available starting materials. PU networks were prepared by incorporating varying amounts of (B) BCS or (C) TCJ CAs into standard industrial PU components, and their deconstructability was evaluated.

To assess the selective cleavability of this CA, we synthesized a dicarbamate model compound, **Et**_**2**_ **Si­(OC**_**6**_ **H**_**12**_ **OC­(O)­NHCy)**_**2**_, and evaluated various conditions to identify the optimal method for cleaving the Si–O bonds while preserving the carbamate linkages. After extensive screening, we were pleased to discover that catalytic FeCl_3_—an abundant, nontoxic, and cost-effective reagent—selectively cleaves the Si–O bonds in a tetrahydrofuran (THF)/methanol (9:1, v/v) mixture at 50 °C. ^1^H NMR analysis of the crude mixture confirmed the quantitative conversion of **Et**_**2**_ **Si­(OC**_**6**_ **H**_**12**_ **OC­(O)­NHCy)**_**2**_ to the hydroxyl-terminated cleavage product, with no evidence of carbamate cleavage or other deleterious reactions (Figure S2). Building on this result, we synthesized a corresponding trifunctional analog, **EtSi­(OC**_**6**_ **H**_**12**_ **OH)**_**3**_, as a TCJ in good yield (79%) using trichloroethylsilane and hexanediol as readily available starting materials (Figure 2A).

### Synthesis and Deconstruction of BCS- and TCJ-Based PU Networks

Using these CAs, we synthesized BCS- and TCJ-based PU thermosets via conventional formulations and evaluated their influence on network deconstruction (Figures 2B and 2C). The formulations comprised standard PU building blocks, including isocyanate-terminated prepolymers derived from poly­(tetrahydrofuran) (pTHF) and hexamethylene diisocyanate (HDI), 1,6-hexanediol as a chain extender, and glycerol as a cross-linker. To determine the critical CA loading required for network deconstruction, we prepared materials with varying BCS and TCJ loadings and compared the experimental results to predictions from our reverse gel-point model.

BCS-based PUs were synthesized over a range of [BCS]/[cross-linker] ratios—spanning values both below and above the predicted critical threshold for deconstruction (1.5, corresponding to *f*_*e*_ values below and above 2). The synthesis commenced with the formation of an isocyanate-terminated prepolymer by coupling poly­(tetrahydrofuran) (pTHF) with hexamethylene diisocyanate (HDI) at 80 °C in the presence of dibutyltin dilaurate (DBTDL) catalyst (Figure 2B). The prepolymer was subsequently combined with glycerol, 1,6-hexanediol, and **Et**_**2**_ **Si­(OC**_**6**_ **H**_**12**_ **OH)**_**2**_ in THF, and the resulting mixtures were cast into molds. The samples were cured at room temperature for 24 h, followed by an additional 12 h cure at 100 °C under vacuum, yielding colorless, flexible materials. Fourier-transform infrared (FTIR) spectroscopy confirmed near-quantitative conversion of HDI under these curing conditions, as evidenced by the absence of isocyanate peaks in the spectra (Figure S5). In total, five BCS-based PU networks were synthesized, each with a fixed cross-linker loading (0.3 equiv glycerol relative to the moles of prepolymer). The number of cleavable functionalities was modulated by adjusting the molar equivalents of BCS relative to the prepolymer, while maintaining a 1:1 alcohol-to-isocyanate ratio across the series by appropriately adjusting the 1,6-hexanediol content. The weight fraction of BCS in these networks ranged from 2.9 to 14.5 wt %.

The resulting BCS-containing PUs were subjected to methanolysis using FeCl_3_ in THF/MeOH (9:1, v/v) at 50 °C for 24 h; their deconstructabilities were evaluated against the model predictions based on their theoretical *f*_*e*_ values. After the reaction, the samples were cooled, the supernatant was removed, and any remaining insoluble fragments were rinsed with excess THF, dried under vacuum, and weighed. As expected, the control sample showed no measurable mass loss under these conditions (Figure 2B). By contrast, samples containing 0.1 equiv (2.9 wt %), 0.2 equiv (5.7 wt %), and 0.3 equiv (8.4 wt %) of **Et**_**2**_ **Si­(OC**_**6**_ **H**_**12**_ **OH)**_**2**_ experienced substantial but incomplete deconstruction, with mass losses of 19%, 38%, and 81%, respectively. Near-complete mass loss (>99%) was observed for samples with 0.4 equiv (11.6 wt %) and 0.5 equiv (14.5 wt %) of **Et**_**2**_ **Si­(OC**_**6**_ **H**_**12**_ **OH)**_**2**_, with no intact material visually detected. Based on these results, the critical BCS loading required to achieve full network deconstruction falls between 0.3 equiv (8.4 wt %) and 0.4 equiv (11.6 wt %). Importantly, the weight-average functionality of −OH groups, *f*_*e*_, is 2.05 at the latter loading, slightly exceeding the critical *f*_*e*_ = 2 predicted by the reverse gel point model. This minor discrepancy between experimental results and theoretical predictions is likely attributed to network defects, such as dangling ends or loops, which are not accounted for in the model but are present in networks.^54–57^

Similarly, four TCJ-based PU thermosets were synthesized following analogous procedures, with a fraction of glycerol replaced by **EtSi­(OC**_**6**_ **H**_**12**_ **OH)**_**3**_ in varying ratios. The number of cleavable bonds was varied by adjusting the molar equivalents of TCJ relative to the moles of prepolymer, while maintaining a 1:1 ratio of alcohol to isocyanate groups by appropriately tuning the glycerol content. The total cross-linker loading (TCJ + glycerol) was consistently held at 0.3 equiv across all formulations. The weight fraction of TCJ in these materials ranged from 1.8 wt % to 7.1 wt %. The materials were subjected to FeCl_3_-catalyzed methanolysis under the same conditions as for the deconstruction of BCS-based networks, and the results were compared to model predictions (Figure 2C).^58,59^ Consistent with our observations for BCS-based networks, samples with the lowest **EtSi­(OC**_**6**_ **H**_**12**_ **OH)**_**3**_ loadings (0.05 equiv, 1.8 wt % and 0.10 equiv, 3.6 wt %) showed incomplete mass loss. In contrast, networks with **EtSi­(OC**_**6**_ **H**_**12**_ **OH)**_**3**_ loadings of 0.15 equiv (5.4 wt %) and 0.20 equiv (7.1 wt %) exhibited near-complete mass loss, with no intact material visually detected. These results establish that the critical TCJ loading required for complete network deconstruction lies between 0.10 equiv (3.6 wt %) and 0.15 equiv (5.4 wt %). Significantly, at the latter TCJ loading, *f*_e_ attains a value of 2.0, in excellent agreement with the reverse gel point model. Furthermore, consistent with model predictions, this TCJ loading (0.15 equiv) is 63% lower than the critical BCS loading (0.4 equiv) on a molar basis and 51% lower by weight.

These findings highlight that BCS and TCJ CAs offer two complementary approaches for achieving the deconstruction of PUs primarily composed of noncleavable polyols and diisocyanates, the minimal loading of which can be predicted by reverse gel-point theory. The incorporation of these CAs has enabled the deconstruction of PU networks derived from aliphatic isocyanates under mild conditions using abundant, low-cost reagents. The latter findings are particularly significant, as aromatic isocyanates are generally considered easier to deconstruct than their aliphatic counterparts, which exhibit greater thermal stability and are more challenging to reprocess.^55,56^ Consequently, many existing PU deconstruction methods are limited to materials synthesized from aromatic isocyanates.^13,26,60^

### Properties of BCS- and TCJ-Based PU Networks

In PU elastomers, thermodynamic immiscibility between the hard and soft segments drives microphase separation, which significantly impacts material properties.^61–66^ The soft segments, typically derived from polyether or polyester polyols, have glass transition temperatures (*T*_g_) well below room temperature, providing flexibility and elasticity.^61–64^ In contrast, the hard segments, composed of diisocyanates and chain extenders, form rigid domains through hydrogen bonding and crystallization, contributing to mechanical strength.^63–66^ The interplay between these segments is crucial in determining the overall properties of PU materials. Given these structural characteristics, we anticipated that incorporating CAs would influence the properties of PU thermosets and may provide handles for property tuning in addition to imparting deconstructability. To investigate these effects, we synthesized a control virgin PU thermoset (**vPU**) and two fully deconstructable PU thermosets (**dPU-BCS**_**0.45**_ and **dPU-TCJ**_**0.15**_), incorporating BCS and TCJ CAs, respectively. Both thermosets were prepared with CA loadings matching the critical thresholds for full network deconstruction predicted by reverse gel-point theory (0.45 equiv for BCS and 0.15 equiv for TCJ, Figure 3A). In **dPU-BCS**_**0.45**_, 82 mol % of the chain extender 1,6-hexanediol was substituted with **Et**_**2**_ **Si­(OC**_**6**_ **H**_**12**_ **OH)**_**2**_, while in **dPU-TCJ**_**0.15**_, 50 mol % of the cross-linker glycerol was replaced with **EtSi­(OC**_**6**_ **H**_**12**_ **OH)**_**3**_. All three materials were synthesized using otherwise identical compositions and curing conditions (see SI for detailed procedures).

**Figure 3:**
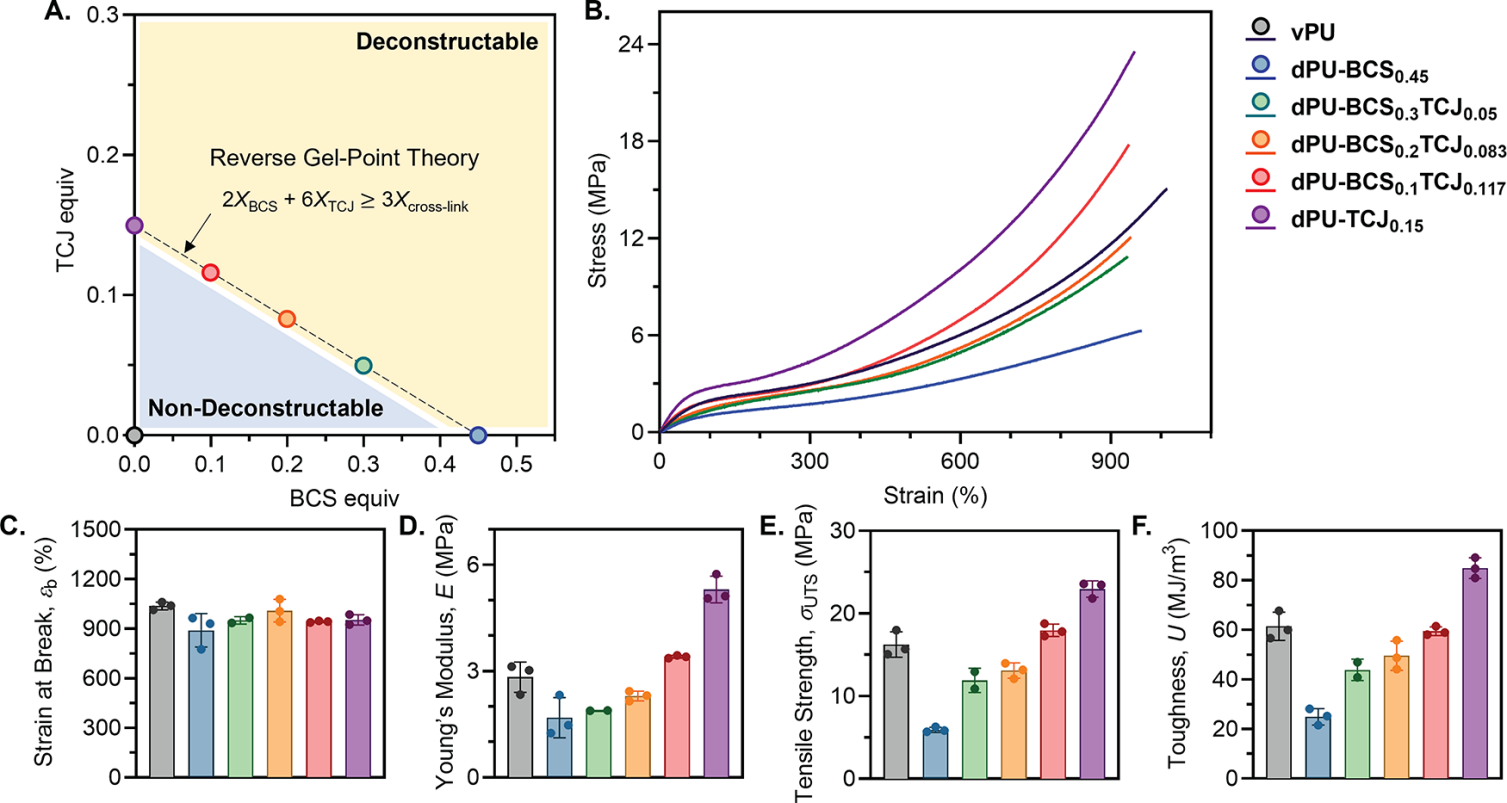
Uniaxial tensile properties of BCS- and TCJ-based PU networks. (A) The BCS and TCJ loadings were determined in accordance with reverse gel-point theory to remain minimal while ensuring complete deconstruction. The coordinates indicated by colored circles on the plot represent the chosen loadings of CAs. (B) Representative stress–strain curves are shown; see the SI for complete replicates. Plots comparing (C) strain at break (*ε*_b_), (D) tensile modulus (*E*), (E) tensile strength (σ_UTS_), and (F) toughness (*U*) are shown. Data for each material can be identified by the corresponding colors in panels A and B.

The tensile properties of **dPU-BCS**_**0.45**_ and **dPU-TCJ**_**0.15**_ were evaluated and compared to those of **vPU** (Figure 3B). While the elongation at break (*ε*_b_) remained largely retained (Figure 3C), differences in tensile moduli (*E*) were observed, following the trend: **dPU-BCS**_**0.45**_ (1.7 ± 0.57 MPa) < **vPU** (2.8 ± 0.43 MPa) < **dPU-TCJ**_**0.15**_ (5.3 ± 0.38 MPa) (Figure 3D). This variation in *E* can be attributed to changes in the effectiveness of hard segment formation within the networks. In **dPU-BCS**_**0.45**_, replacing 1,6-hexanediol with **Et**_**2**_ **Si­(OC**_**6**_ **H**_**12**_ **OH)**_**2**_ likely disrupts the formation of rigid domains, leading to a lower modulus and ultimate tensile strength (σ_UTS_: 5.9 ± 0.31 MPa) compared to **vPU** (16.3 ± 1.54 MPa) and **dPU-TCJ**_**0.15**_ **(**23.0 ± 0.98 MPa) (Figure 3E). Conversely, in **dPU-TCJ**_**0.15**_, substituting glycerol with the more conformationally flexible **EtSi­(OC**_**6**_ **H**_**12**_ **OH)**_**3**_ likely promotes hard segment formation by increasing chain mobility, thereby enhancing interchain interactions and packing. This increased flexibility is supported by dynamic mechanical analysis (DMA), which showed a lower glass transition temperature (*T*_g,DMA_) for **dPU-TCJ**_**0.15**_ (−39 ± 1.4 °C) compared to **vPU** (−28 ± 0.6 °C) and **dPU-BCS**_**0.45**_ (−30 ± 1.8 °C) (Figures S16–18). The trend in toughness (*U*) followed the same pattern as *E* and σ_UTS_: **dPU-BCS**_**0.45**_ (25 ± 3.3 MJ/m^3^) < **vPU** (61 ± 5.7 MJ/m^3^) < **dPU-TCJ**_**0.15**_ (85 ± 4.1 MJ/m^3^) (Figure 3F). The addition of **Et**_**2**_ **Si­(OC**_**6**_ **H**_**12**_ **OH)**_**2**_ and **EtSi­(OC**_**6**_ **H**_**12**_ **OH)**_**3**_ did not substantially compromise the thermal stability of PU thermosets, as evidenced by thermogravimetric analysis (TGA), which showed 5% mass loss at 255 °C for **dPU-BCS**_**0.45**_ and 271 °C for **dPU-TCJ**_**0.15**_, compared to 268 °C for **vPU** (Figures S22–24).

We anticipated that the opposing effects of **Et**_**2**_ **Si­(OC**_**6**_ **H**_**12**_ **OH)**_**2**_ and **EtSi­(OC**_**6**_ **H**_**12**_ **OH)**_**3**_ on the mechanical properties of PU thermosets, combined with our predictive reverse gel point model (eq 2), which is applicable to mixtures of BCS and TCJ CAs, would enable the design of fully deconstructable PU thermosets with tunable material properties. To test this hypothesis, we synthesized three thermosets with a mixed introduction of **Et**_**2**_ **Si­(OC**_**6**_ **H**_**12**_ **OH)**_**2**_ and **EtSi­(OC**_**6**_ **H**_**12**_ **OH)**_**3**_—**dPU-BCS**_**0.3**_ **TCJ**_**0.05**_, **dPU-BCS**_**0.2**_ **TCJ**_**0.083**_, and **dPU-BCS**_**0.1**_ **TCJ**_**0.117**_—where the subscripts indicate the equivalents of each introduced. All thermosets were prepared with CA loadings matching the critical thresholds for full network deconstruction predicted by reverse gel-point theory (Figure 3A).

As predicted, **dPU-BCS**_**0.3**_ **TCJ**_**0.05**_, **dPU-BCS**_**0.2**_ **TCJ**_**0.083**_, and **dPU-BCS**_**0.1**_ **TCJ**_**0.117**_ were fully deconstructable under identical FeCl_3_-catalyzed methanolysis conditions (Figure S28). Uniaxial tensile testing on these materials, along with a comparison to the results for **dPU-BCS**_**0.45**_ and **dPU-TCJ**_**0.15**_ revealed a general trend (Figure 3B): while *ε*_b_ remained largely consistent throughout the series (Figure 3C), increased **EtSi­(OC**_**6**_ **H**_**12**_ **OH)**_**3**_ loading combined with a corresponding decrease in **Et**_**2**_ **Si­(OC**_**6**_ **H**_**12**_ **OH)**_**2**_ loading resulted in increases of *E*, σ_UTS_, and *U* (Figures 3D to F), confirming tunability of material properties. The properties of **dPU-BCS**_**0.2**_ **TCJ**_**0.083**_ and **dPU-BCS**_**0.1**_ **TCJ**_**0.117**_ closely resembled those of **vPU**, demonstrating that the CA approach can preserve mechanical properties while introducing full deconstructability. We highlight that the synthesis of these materials requires replacing only 8.6 and 7.0 wt % of the conventional components for industrial PU synthesis, respectively.

### Chemical Recycling of TCJ-Based PUs

Finally, we examined the recyclability of fragments collected from the deconstruction of CA-embedded PU thermosets. As a representative example, we focused on fragments obtained from the deconstruction of TCJ-based thermosets. **dPU-TCJ-G0** was prepared with 0.2 equiv **EtSi­(OC**_**6**_ **H**_**12**_ **OH)**_**3**_ and deconstructed under FeCl_3_-catalyzed methanolysis conditions. The resulting fragments were purified through a simple aqueous extraction to remove FeCl_3_, yielding an 81% mass recovery (Figure S3, see SI for detailed procedure). Size-exclusion chromatography (SEC) analysis of the purified fragments revealed a number-average molar mass (*M*_n_) of ∼5.6 kDa (Figure S29), which is in close agreement with the theoretically derived value of 4.5 kDa (see SI for details). We next quantified the hydroxyl content of the purified fragments using ^1^H NMR. For precise measurement, the fragments were exposed to trifluoroacetic anhydride to form trifluoroacetate esters, whose well-resolved α proton signals were used for the quantification (Figure S4, see SI for detailed procedure).

To synthesize the deconstructable, recycled PU thermoset, 35 mol % of 1,6-hexanediol in the original **dPU-TCJ-G0** formulation was substituted with the purified fragments. The molar proportions of the prepolymer remained unchanged, while glycerol was entirely substituted with **EtSi­(OC**_**6**_ **H**_**12**_ **OH)**_**3**_ at 0.30 equiv relative to the prepolymer (Figure 4A). Based on this formulation, a first-generation recycled PU thermoset, **rPU-TCJ-G1­(38)**, was synthesized with 38 wt % **dPU-TCJ-G0** fragments and 6.5 wt % **EtSi­(OC**_**6**_ **H**_**12**_ **OH)**_**3**_. The successful synthesis was confirmed by FTIR spectroscopy, which showed the complete conversion of isocyanates (Figure S5). Tensile testing of **rPU-TCJ-G1­(38)** showed an increased modulus (14.0 ± 1.36 MPa) compared to **dPU-TCJ-G0** (9.9 ± 0.43 MPa) (Figure 4B). This increase is likely due to the high 1,6-hexanediol content in the **dPU-TCJ-G0** fragments, which enriches the hard segments and introduces physical cross-links upon recycling (each deconstructed **EtSi­(OC**_**6**_ **H**_**12**_ **OH)**_**3**_ unit generates three 1,6-hexanediol units). The recycled **rPU-TCJ-G1­(38)** exhibited a σ_UTS_ of 14.6 ± 0.52 MPa and a *ε*_b_ of 910 ± 60%, both statistically indistinguishable from **dPU-TCJ-G0**, and a slightly higher *U* of 85 ± 4.7 MJ/m^3^. Additionally, **rPU-TCJ-G1­(38)** showed excellent thermal stability, with a 5% mass loss at 265 °C, comparable to **dPU-TCJ-G0** (Figure S25). These results confirm the successful recycling of **dPU-TCJ-G0** with no loss in its original mechanical properties and thermal stability.

**Figure 4:**
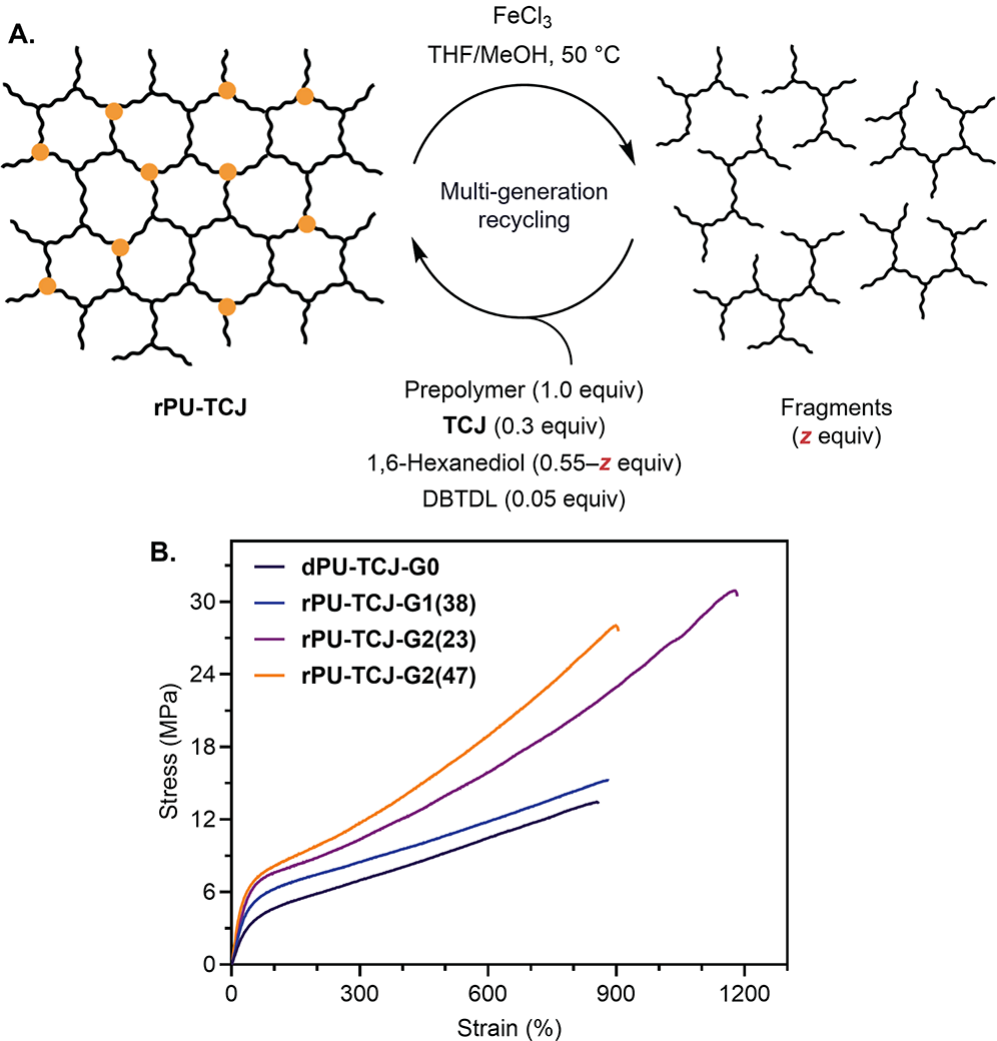
Chemical recycling of TCJ-based PU networks. (A) Schematic of the recycling process and (B) uniaxial tensile properties of the recycled materials. Representative stress–strain curves are shown; see the SI for complete replicates.

To demonstrate multigeneration recyclability, the first-generation recycled material, **rPU-TCJ-G1­(38)**, was fully deconstructed into soluble fragments under FeCl_3_-mediated methanolysis conditions, achieving an 85% mass recovery post-purification. Following hydroxyl end-group quantification, these fragments were reintegrated into the next-generation recycled materials by substituting (1) 35 mol % and (2) 100 mol % of 1,6-hexanediol in the original **dPU-TCJ-G0** formulation. The molar proportions of all other components remained unchanged from the **rPU-TCJ-G1­(38)** (Figure 4A). This process yielded **rPU-TCJ-G2­(23)** and **rPU-TCJ-G2­(47)**, containing 23 and 47 wt % **rPU-TCJ-G1­(38)** fragments, respectively, along with 8.1 and 5.9 wt % **EtSi­(OC**_**6**_ **H**_**12**_ **OH)**_**3**_. Both **rPU-TCJ-G2­(23)** and **rPU-TCJ-G2­(47)** exhibited improved mechanical properties (Figure 4B), with higher *E* (20.3 ± 2.92 MPa and 22.1 ± 1.24 MPa, respectively) and σ_UTS_ (30.7 ± 0.45 MPa and 27.8 ± 0.59 MPa, respectively), as well as higher or comparable *ε*_b_ (1190 ± 92% and 920 ± 15%, respectively) compared to **rPU-TCJ-G1­(38)**. These improvements resulted in enhanced *U* (198 ± 4.7 MJ/m^3^ and 140 ± 4.7 MJ/m^3^, respectively), while *T*_g,DMA_ remained consistent, at −43 ± 0.6 °C and −42 ± 1.3 °C, compared to −44 ± 1.3 °C for **rPU-TCJ-G1­(38)** (Figures S19–21). The second-generation recycled materials also exhibited 5% mass loss measured by TGA at 270 and 271 °C, closely matching the thermal stability of **vPU**, **dPU-TCJ-G0** and **rPU-TCJ-G1­(38)** (Figures S22, 25–27). Notably, both **rPU-TCJ-G2­(23)** and **rPU-TCJ-G2­(47)**, featuring enhanced mechanical properties and retained thermal stability relative to earlier-generation materials, underwent full deconstruction under identical methanolysis conditions, yielding soluble fragments (Figure S28). These results highlight their potential for further recyclability. We note that this method may not support indefinite recycling, as successive generations yield increasingly high-molar-mass species (Figure S30).

## Conclusion

In conclusion, we introduced two strategies that employ Si–O-based CAs to render PU thermosets—composed primarily of conventional components—fully deconstructable into soluble products under mild, selective conditions. We developed a predictive model that accurately determines the minimum CA loading required for complete deconstruction at any cross-linker concentration, facilitating a comparative analysis of the BCS and TCJ approaches. While both strategies effectively enabled network deconstruction, the TCJ approach proved more efficient, achieving full deconstructability at lower CA loadings (∼5 wt % vs ∼ 12 wt % for BCS). Furthermore, we demonstrated that the combined use of both CA types enables strategic control over PU mechanical properties, allowing either their preservation or deliberate modification. The soluble fragments obtained from deconstruction were subsequently recycled into new PU thermosets without compromising their thermal and mechanical properties over two deconstruction/reconstruction cycles. This demonstration confirms the feasibility of synthesizing multiple generations of recyclable PU materials without altering standard manufacturing workflows or replacing key network components. Overall, this work underscores the potential of CAs within step-growth networks and may represent a practical advancement toward the commercial-scale production of deconstructable and recyclable PU thermosets. Looking ahead, introducing alternative cleavable motifs or end groups into the BCS and TCJ frameworks could extend this strategy to more demanding environments (e.g., prolonged acidic conditions) and diverse polymer networks. Future efforts should focus on raising recycled content to approach 100%, alongside developing deconstruction protocols that minimize solvent and reagent use for more effective implementation.

## Supplementary Material



## Notes

Materials and analytical methods employed, details of the reverse gel-point theory, comprehensive experimental procedures, and additional characterization data (PDF)
