# Supplementary material for: Cleavable Additives for Deconstructable, Recyclable Polyurethane Thermosets
Source: ACS Cent Sci. 2025 Jul 23;11(8):1355–63. doi: 10.1021/acscentsci.5c00689 (PMC12395304; doi:10.1021/acscentsci.5c00689)
Supplement: Supplementary file 1 [file oc5c00689_si_001.pdf]

Supporting Information for:

Cleavable Additives for Deconstructable, Recyclable Polyurethane Thermosets

Kwangwook Ko[1], David J. Lundberg[2], Valerie L. Lensch[1], Yasmeen S. AlFaraj[1], Keith E. L. Husted[1], Jacob P. Brutman[3], Alaaeddin Alsbaiee[3], Patrick N. Hamilton[3], Suong T. Nguyen[1]*, and Jeremiah A. Johnson[1]*

Affiliations

[1]Department of Chemistry, Massachusetts Institute of Technology, 77 Massachusetts Avenue, Cambridge, MA 02139, U.S.A.

[2]Department of Chemical Engineering, Massachusetts Institute of Technology, 25 Ames St, Cambridge, MA 02139, U.S.A.

[3]BASF Corporation, 1609 Biddle Avenue, Wyandotte, MI 48192, U.S.A

*Correspondence to: jaj2109@mit.edu

Table of Contents

1. General Experimental Details……S3

1.1. General Materials Information……S3

1.2. General Analytical Information……S3

2. Reverse Gel-Point Theory……S5

2.1. General Considerations……S5

2.2. Derivation of Equations for PU Networks……S6

2.3. Effects of Off-Stoichiometry……S8

3. Experimental Procedures……S9

3.1. Synthesis of Monomers……S9

3.2. Model Experiments……S10

3.3. Synthesis of Polyurethane Networks……S12

3.4. Network Deconstruction……S14

3.5. Chemical Recycling……S17

4. Supplementary Data……S19

4.1. Fourier-Transform Infrared Spectroscopy (FTIR)……S19

4.2. Tensile Testing……S20

4.3. Dynamic Mechanical Analysis (DMA)……S25

4.4. Thermogravimetric Analysis (TGA)……S28

4.5. Deconstruction Experiments……S31

4.6. Size Exclusion Chromatography (SEC)……S32

4.7. Nuclear Magnetic Resonance Spectroscopy (NMR)……S35

5. References……S38

1. General Information

1.1. General Materials Information

Unless otherwise noted, all reagents were purchased from commercial vendors and used as received. Solvents used for synthesis were of reagent grade and were used without any further purification. HPLC grade solvents were used for spectroscopic studies. Anhydrous tetrahydrofuran (THF), chloroform, dichloromethane (CH_2Cl_2), and triethylamine (TEA) were purchased from Millipore-Sigma packaged in Sure/Seal™ bottles and used as received. Poly(tetrahydrofuran) (pTHF) was dried over anhydrous sodium sulfate and magnesium sulfate prior to use. Organic solutions were concentrated under reduced pressure on a Büchi rotary evaporator. All reactions were carried out under ambient conditions in well ventilated fume hoods unless otherwise noted. Thin-layer chromatography (TLC) was performed on Silicycle 250 µm silica gel plates. Visualization of the developed chromatogram was performed by irradiation with UV light or treatment with a solution of potassium permanganate stain followed by drying. Normal-phase flash chromatography was performed using a Biotage Isolera One purification system equipped with a 10, 25, 50, or 100 g Biotage Duo cartridge and an appropriate linear gradient in the mobile phase. Yields refer to purified compounds unless otherwise noted.

1.2. General Analytical Information

1.2.1. Nuclear magnetic resonance spectroscopy (NMR).

^1H and ^{13}C NMR spectra were collected using either Bruker AVANCE III DRX 400 or Neo 500 spectrometer at 25 °C and were internally referenced to residual solvent signals: $CDCl_3$ referenced at δ 7.26 (^1H) and 77.16 ppm (^{13}C). Data for ^1H are reported as follows: chemical shift (δ, ppm), integration, multiplicity (s = singlet, d = doublet, t = triplet, q = quartet, p = quintet, h = sextet, m = multiplet), broad peaks (br), coupling constant (Hz) and assignment. Data for ^{13}C NMR are reported in terms of chemical shift and no special nomenclature is used for equivalent carbons.

1.2.2. High-resolution mass spectrometry (HR-MS)

High resolution mass spectrometry (HR-MS) measurements were recorded using a JEOL AccuTOF 4G LC-plus system equipped with an ionSense Direct Analysis in Real Time (DART) source.

1.2.3. Size exclusion chromatography (SEC)

Analytical size exclusion chromatography (SEC) was performed in HPLC-grade THF using an Agilent 1260 Infinity system, equipped with an Agilent PLgel guard column (5 µm; 50 x 7.5 mm) and three Agilent PLgel analytical columns (5 µm; 300 x 7.5 mm; 105, 104, and 103 Å pore sizes). The analysis was conducted at 35 °C with a flow rate of 1.0 mL/min. The instrument was calibrated with polystyrene standards. Molecular weight and dispersity values

were calculated using ChemStation GPC Data Analysis Software (rev. B.01.01), based on the refractive index signal.

1.2.4. Thermogravimetric analysis (TGA)

Thermogravimetric analysis was carried out on samples of approximately 2-5 mg. The analyses were conducted using a TGA/DSC 2 STAR System (Mettler-Toledo) equipped with a Gas Controller GC 200 Star System. The studies were carried out under a constant stream of nitrogen gas at a temperature ramp of 10 °C/min. Temperature ranges from 30 to 800 °C. Experiments were performed at the MIT Institute for Soldier Nanotechnologies.

1.2.5. Dynamic mechanical analysis (DMA)

DMA was performed on a TA Instruments DMA Q850 at the MIT Institute for Soldier Nanotechnologies. Samples were tested in tension mode. Measurements were recorded at a frequency of 1.0 Hz and an amplitude of 10.0 μm from −50 °C to 50 °C at a ramp rate of 3 °C/min with a data sampling interval of 3 s/pt, using a 125% force tracking and 0.01 N preload force. A TA instruments Liquid Nitrogen Purge Cooler (NPC) was used for samples which required low temperature measurements. Data were collected using Trios software and exported to Prism for analysis.

1.2.6. Tensile testing

Dogbone samples were prepared with 0.25 scale D412C tensile sample cutting die from Fremont Cutting Dies Inc. Tensile testing was performed on an 8848 MicroTester (Instron) with an extension rate of 0.5 mm/s, using a 2 kN load cell with a preload force of 1N. Experiments were performed at the MIT Department of Materials Science and Engineering Nano Mechanical Technology Laboratory.

2. Reverse Gel-Point Theory

2.1. General Considerations

The critical loading of cleavable strands or junctions required for network deconstruction can be predicted using reverse gel-point model, derived from the Miller-Macosko theory.[1, 2] This approach assesses network deconstructability by analyzing the assembly of fragment components generated from the cleavage of labile bonds within the network components and determining whether they are capable of assembling to form a percolated gel. If the fragment components do not satisfy the percolation threshold indicated by Miller-Macosko theory, the cleavable additive-containing network is deemed deconstructable. Non-percolation condition at quantitative conversion can be defined as reverse gel-point.

In the general case of two-component step-growth network formation between components “A" and “B”, the percolation condition is defined by:[1,2]

$$rp_{gel}^2 > \frac{1}{(f_e - 1)(g_e - 1)} \tag{S1}$$

where r is the stoichiometric ratio (always defined to be <1), p_{gel} is the extent of reaction at the percolation threshold, and f_e and g_e are the effective average functionalities of A and B network components, respectively. For mixtures of components with different functionalities, these effective average functionality values are first determined by calculating the mole fraction of the A or B reactive groups on each different functionality component, a_{f_i} and b_{g_j}, respectively:

$$a_{f_i} = \frac{f_i A_{f_i}}{\sum_i f_i A_{f_i}} \tag{S2}$$

$$b_{g_j} = \frac{g_j B_{g_j}}{\sum_j g_j B_{g_j}} \tag{S3}$$

where A_{f_i} and B_{g_j} are the molar equivalents of components with functionalities f_i and g_j, respectively. The values of f_e and g_e are then calculated as a functionality-weighted average as follows:

$$f_e = \sum_i f_i a_{f_i} \tag{S4}$$

$$g_e = \sum_j f_j b_{g_j} \tag{S5}$$

2.2. Derivation of Equations for PU Networks

In a PU network formed from diisocyanate (hexamethylene diisocyanate, HMDI), diols (1,6-hexanediol), triols (glycerol), BCSs, and TCJs as network components, the fragment components are as illustrated in Figure S1.

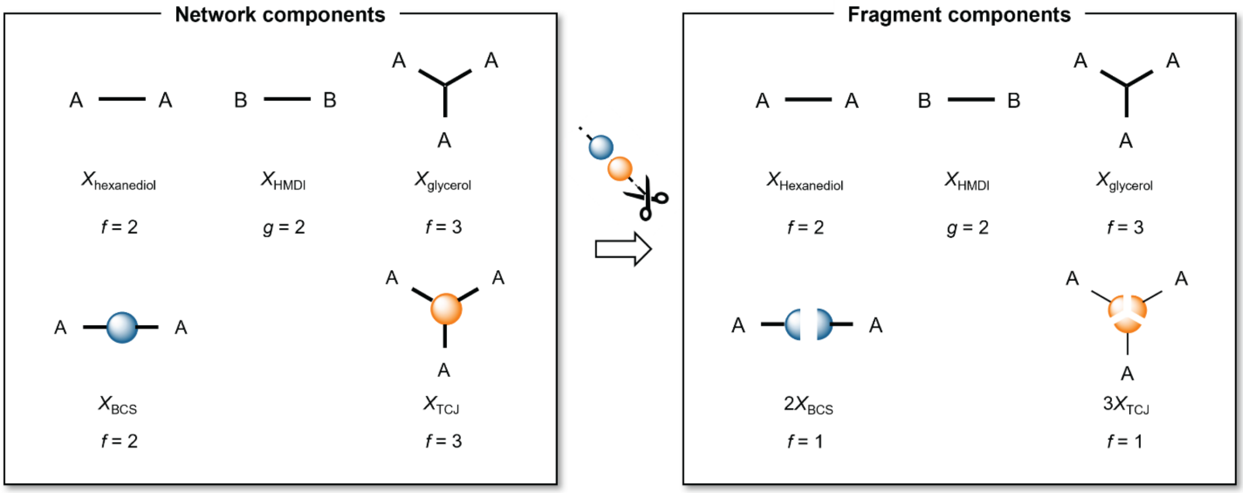

Figure S1. Analysis of fragment components for the evaluation of reverse gel-point

For networks formulated on stoichiometry (i.e., equimolar amounts of alcohol and isocyanate functionalities) the stoichiometric ratio, *r*, is 1. The exclusive use of bifunctional isocyanate components sets g_e equal to 2. Applying these values simplifies the percolation condition given by Equation S1 at complete conversion:

$$p = 1 > \sqrt{\frac{1}{(f_e - 1)}} \tag{S6}$$

Deconstructability is predicted when BCS cleavage yields a network below the percolation threshold, which occurs when:

$$f_e \leq 2 \tag{S7}$$

From equation S4, the effective functionality is defined as:

$$f_e = 1a_1 + 2a_2 + 3a_3 \leq 2 \tag{S8}$$

with the constraint:

$$a_1 + a_2 + a_3 = 1 \tag{S9}$$

Thus, satisfying equation S8 leads to the percolation condition being met when

$$a_1 \geq a_3 \tag{S10}$$

In our system of interest (Figure S1), the following relationships hold:

$$A_1 = 2X_{BCS} + 3X_{TCJ} \tag{S11}$$

$$A_2 = X_{hexanediol} \tag{S12}$$

$$A_3 = X_{glycerol} \tag{S13}$$

Therefore, a_1 and a_3 are expressed as:

$$a_1 = \frac{A_1}{A_1 + 2A_2 + 3A_3} = \frac{2X_{BCS} + 3X_{TCJ}}{2X_{BCS} + 3X_{TCJ} + 2X_{hexanediol} + 3X_{glycerol}} \tag{S14}$$

$$a_3 = \frac{3A_3}{A_1 + 2A_2 + 3A_3} = \frac{3X_{glycerol}}{2X_{BCS} + 3X_{TCJ} + 2X_{hexanediol} + 3X_{glycerol}} \tag{S15}$$

Substituting equations S14 and S15 into equation S10 yields:

$$2X_{BCS} + 3X_{TCJ} \geq 3X_{glycerol} \tag{S16}$$

Since glycerol and TCJ function as cross-linkers in the network, we define the molar amount of cross-linking component as:

$$X_{cross-link} = X_{glycerol} + X_{TCJ} \tag{S17}$$

Substituting this definition into equation S16 leads to:

$$2X_{BCS} + 6X_{TCJ} \geq 3X_{cross-link} \tag{S18}$$

2.3. Effects of Off-Stoichiometry

The above analysis considered the case when the stoichiometric ratio is equal to 1. In practice, advantageous water or other impurities may react preemptively with isocyanate functionalities, pushing r to be less than 1. In this case the effective functionality of isocyanate components, g_e, takes a value less than 2. By inspection of Equation S1, we see that values of r less than 1 will *decrease* the magnitude of the left-hand side of the equation, and g_e values less than 2 will *increase* the magnitude of the right-hand side of the equation. Both deviations work in the same 'direction' making network formation more difficult, i.e., the value of p_{gel} must be greater to account for these changes. In the context of predicting network deconstruction (i.e., rearranging Equation S1. with p = 1), these deviations lead to a *lower* critical functionality for deconstruction (less than 2). Overall, networks formed off-stoichiometry are predicted to require fewer BCSs or TCJs to be deconstructed.

3. Experimental Procedures

3.1. Synthesis of Monomers

3.1.1. Synthesis of **$Et_2Si(OC_6H_{12}OH)_2$**

To a 500-mL flame-dried round-bottom flask equipped with a stir bar was added hexanediol (35.5 g, 300 mmol, 20 equiv). The mixture was degassed and backfilled with nitrogen (3 times) before anhydrous CH_2Cl_2 (250 mL) and triethylamine (4.4 mL, 31.5 mmol, 2.1 equiv) were added. Dichlorodiethylsilane (2.2 mL, 15 mmol, 1.0 equiv) was added to an addition funnel containing 40 mL anhydrous CH_2Cl_2, and this solution was added dropwise to the reaction mixture. After the addition was complete, the reaction was allowed to stir for an additional 1 h. The reaction was washed with deionized water (300 mL x 5) and brine. The organic layer was dried with magnesium sulfate for 1 h and solvent was removed under reduced pressure. The crude product was purified via column chromatography (silica gel, hexanes/acetone, 0 to 80% acetone) to give the pure product as a colorless oil (2.51 g, 52% yield).

^{1}H NMR (400 MHz, $CDCl_3$) δ 3.71 (t, *J* = 6.6 Hz, 4H), 3.67 (t, *J* = 6.6 Hz, 4H), 1.64 – 1.56 (m, 8H), 1.41 (h, *J* = 2.8 Hz, 8H), 0.99 (t, *J* = 7.9 Hz, 6H), 0.63 (q, *J* = 8.0 Hz, 4H).

^{13}C NMR (101 MHz, $CDCl_3$) δ 63.05, 62.64, 32.89, 32.79, 25.76, 25.68, 6.66, 3.88.

HR-MS (DART-TOF): $[M–H]^-$ ($C_{16}H_{35}O_4Si$) calcd: 319.22991 m/z, found 319.23179 m/z

3.2.2. Synthesis of **$EtSi(OC_6H_{12}OH)_3$**

To a 500-mL flame-dried round-bottom flask equipped with a stir bar was added hexanediol (32.5 g, 275 mmol, 45 equiv) and imidazole (1.87 g, 27.5 mmol, 4.5 equiv). The mixture was degassed and backfilled with nitrogen (3 times) before anhydrous CH_2Cl_2 (300 mL) was added. Trichloroethylsilane (0.8 mL, 6.1 mmol, 1.0 equiv) was added to an addition funnel containing 40 mL anhydrous CH_2Cl_2, and this solution was added dropwise to the reaction mixture. After the addition was complete, the reaction was allowed to stir for an additional 1 h. The reaction was washed with deionized water (300 mL x 5) and brine. The organic layer was dried with magnesium sulfate for 1 h and solvent was removed under reduced pressure to give the pure product as a colorless oil (1.98 g, 79% yield).

^{1}H NMR (400 MHz, $CDCl_3$) δ 3.76 (t, *J* = 6.6 Hz, 6H), 3.66 (t, *J* = 6.6 Hz, 6H), 1.60 (t, *J* = 6.7 Hz, 12H), 1.48 – 1.30 (m, *J* = 7.0, 6.1 Hz, 12H), 1.01 (t, *J* = 7.9 Hz, 3H), 0.63 (q, *J* = 7.9 Hz, 2H).

^{13}C NMR (126 MHz, $CDCl_3$) δ 63.01, 62.78, 32.87, 32.60, 25.70, 25.62, 6.69, 2.26.

HR-MS (DART-TOF): $[M+H]^+$ ($C_{20}H_{45}O_6Si$) calcd: 409.29854 m/z, found: 409.29853 m/z

3.2. Model Experiments

3.2.1. Synthesis of model compound **$Et_2Si(OC_6H_{12}OC(O)NHCy)_2$**

To a flame-dried 25-mL round-bottom flask equipped with a stir bar was added **$Et_2Si(OC_6H_{12}OH)_2$** (641.1 mg, 2.0 mmol, 1.0 equiv), and the mixture was degassed and refilled with N_2 three times. Anhydrous dioxane (10 mL) and dibutyltin dilaurate (24 μL, 40 μmol, 0.02 equiv) were added, followed by the dropwise addition of cyclohexyl isocyanate (0.54 mL, 4.2 mmol, 2.1 equiv). The reaction was allowed to stir at 80 °C under N_2 for 12 h. The reaction mixture was diluted and extracted with DCM and the organic layer was washed with deionized water and brine, dried over sodium sulfate, and concentrated. The crude product was purified via column chromatography (silica gel, hexanes/acetone, 0 to 80% acetone) to give the pure product as a white solid (727 mg, 64% yield).

^{1}H NMR (400 MHz, $CDCl_3$) δ 4.59 (d, *J* = 8.5 Hz, 2H), 4.05 (t, *J* = 6.6 Hz, 4H), 3.69 (t, *J* = 6.6 Hz, 4H), 3.57 – 3.23 (m, 2H), 2.03 – 1.83 (m, 4H), 1.74 (d, *J* = 4.0 Hz, 4H), 1.66 – 1.51 (m, 10H), 1.43 – 1.26 (m, 12H), 1.15 (pd, *J* = 13.5, 3.4 Hz, 6H), 0.99 (t, *J* = 7.9 Hz, 6H), 0.62 (q, *J* = 8.0 Hz, 4H).

^{13}C NMR (126 MHz, $CDCl_3$) δ 156.08, 64.77, 62.61, 49.85, 33.61, 32.74, 29.23, 25.89, 25.69, 25.65, 24.95, 6.67, 3.87.

HR-MS (DART-TOF): $[M+H]^+$ ($C_{30}H_{59}N_2O_6Si$) calcd: 571.41369 m/z, found: 571.41521 m/z

3.2.2. $FeCl_3$-mediated methanolysis

To each 1-dram vial was added the dicarbamate **$Et_2Si(OC_6H_{12}OC(O)NHCy)_2$** (14.3 mg, 0.025 mmol, 1.0 equiv) and a small stir bar. A stock solution of $FeCl_3$ in MeOH was prepared and an appropriate quantity corresponding to 0.2, 0.5, and 1.0 equiv was added to each reaction mixture. The reaction was allowed to stir at 50 °C. After 12 h, the mixture was diluted with CH_2Cl_2 and washed with water (3 times). The organic layer was dried over sodium sulfate and filtered. The solution was concentrated under reduced pressure, and the resulting crude product was subjected to NMR analysis.

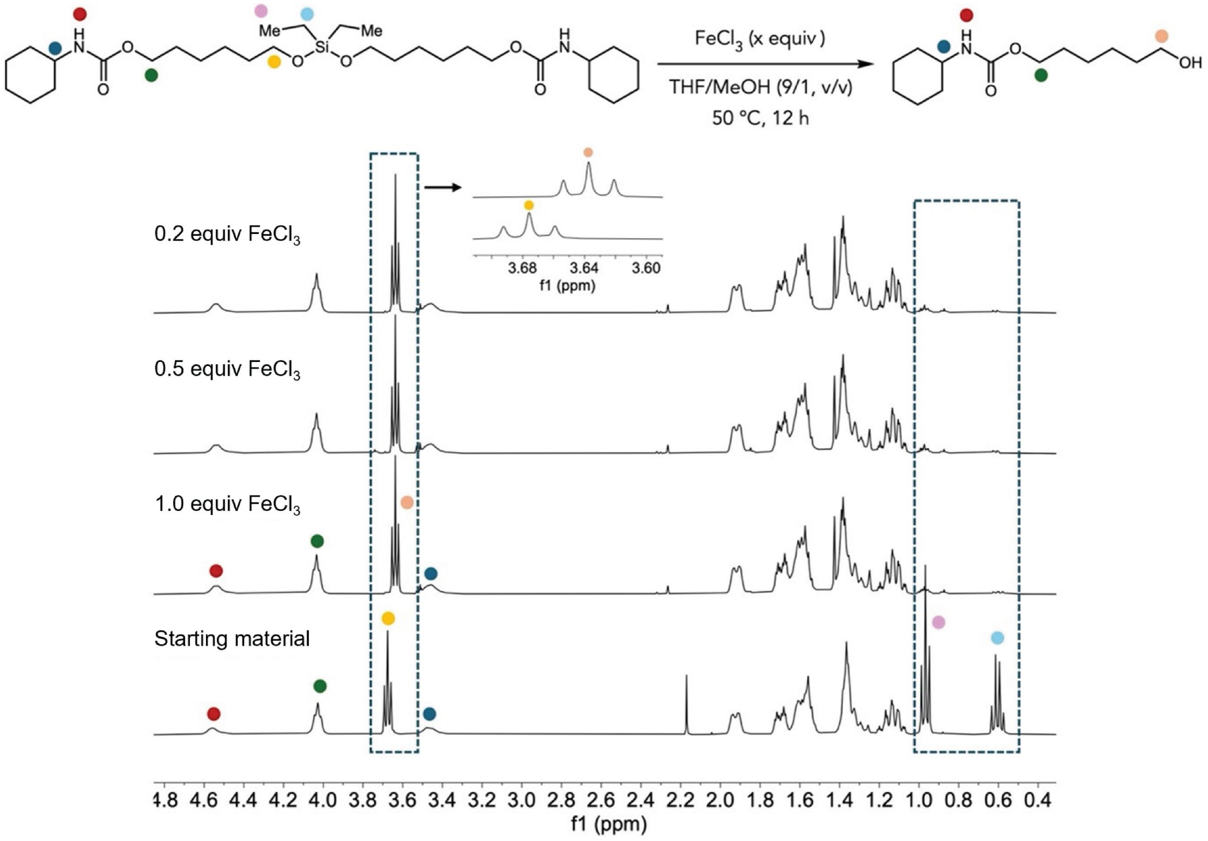

Figure S2. NMR spectra showing the fragmentation reaction of the dicarbamate. The absence of signals at ~0.6 and 0.9 ppm, along with the shift of the signal at ~3.7 ppm, indicates the cleavage of Si–O bonds. In contrast, the unchanged signals at ~3.5, 4.0, and 4.5 ppm suggest that the carbamate bonds remain intact under these conditions.

3.3. Synthesis of Polyurethane Networks

3.3.1. General procedure A

1.755 g of polyTHF (MW = 650, 2.7 mmol, 1.0 equiv) and 908 mg of HMDI (5.4 mmol, 2.0 equiv) were added to a 40-mL vial containing a magnetic stir bar inside a glovebox, and the mixture was stirred thoroughly. The vial was then sealed with a penetrable cap and electrical tape and removed from the glovebox. Using a syringe, 80 μL of DBTDL (0.135 mmol, 0.05 equiv) was added, and the mixture was vortexed. The mixture was subsequently stirred at 80 °C for 2 hours to generate the prepolymer. The vial was returned to the glovebox, and the prepolymer was diluted with 8 mL of dry THF. Separately, 1,6-hexanediol, glycerol, and either **$Et_2Si(OC_6H_{12}OH)_2$** or **$EtSi(OC_6H_{12}OH)_3$** were weighed into individual 4-mL vials (see below for stoichiometries and amounts). The components were dissolved and combined in a total of 3 mL of dry THF, and the resulting solution was transferred into a 6-mL syringe. While vortexing, the 3 mL solution containing 1,6-hexanediol, glycerol, and **$Et_2Si(OC_6H_{12}OH)_2$** or **$EtSi(OC_6H_{12}OH)_3$** was added to the 40 mL vial containing the prepolymer. An additional 1 mL of dry THF was used to rinse the syringe to ensure complete transfer. The resulting mixture was vortexed for approximately 20 seconds, after which it was poured into a Teflon mold. The solvent was allowed to evaporate slowly in the glovebox for 24 hours, forming a uniform polyurethane film. The film was then further dried overnight in a 100 °C vacuum oven.

Table S1. Stoichiometries and Amounts of Reagents for BCS-based PU Networks

	1,6-Hexanediol (mg / mmol / equiv)	**$Et_2Si(OC_6H_{12}OH)_2$** (mg / mmol / equiv)	Glycerol (mg / mmol / equiv)	**$EtSi(OC_6H_{12}OH)_3$** (mg / mmol / equiv)
Control	175.5 / 1.485 / 0.55	–	74.6 / 0.81 / 0.30	–
1	143.6 / 1.215 / 0.45	86.5 / 0.27 / 0.10	74.6 / 0.81 / 0.30	–
2	111.7 / 0.945 / 0.35	173.1 / 0.54 / 0.20	74.6 / 0.81 / 0.30	–
3	79.8 / 0.675 / 0.25	259.6 / 0.81 / 0.30	74.6 / 0.81 / 0.30	–
4	47.9 / 0.405 / 0.15	346.2 / 1.08 / 0.40	74.6 / 0.81 / 0.30	–
5	16.0 / 0.135 / 0.05	432.7 / 1.35 / 0.50	74.6 / 0.81 / 0.30	–

Table S2. Stoichiometries and Amounts of Reagents for TCJ-based PU Networks

	1,6-Hexanediol (mg / mmol / equiv)	$Et_2Si(OC_6H_{12}OH)_2$ (mg / mmol / equiv)	Glycerol (mg / mmol / equiv)	$EtSi(OC_6H_{12}OH)_3$ (mg / mmol / equiv)
Control	175.5 / 1.485 / 0.55	–	74.6 / 0.81 / 0.30	–
1	175.5 / 1.485 / 0.55	–	62.2 / 0.675 / 0.25	55.2 / 0.135 / 0.05
2	175.5 / 1.485 / 0.55	–	49.7 / 0.540 / 0.20	110.3 / 0.270 / 0.10
3	175.5 / 1.485 / 0.55	–	37.3 / 0.405 / 0.15	165.5 / 0.405 / 0.15
4	175.5 / 1.485 / 0.55	–	24.9 / 0.270 / 0.10	220.7 / 0.540 / 0.20

3.3.2. General procedure B

1.755 g of polyTHF (MW = 650, 2.7 mmol, 1.0 equiv) and 908 mg of HMDI (5.4 mmol, 2.0 equiv) were added to a 40-mL vial containing a magnetic stir bar inside a glovebox, and the mixture was stirred thoroughly. The vial was then sealed with a penetrable cap and electrical tape and removed from the glovebox. Using a syringe, 80 μL of DBTDL (0.135 mmol, 0.05 equiv) was added, and the mixture was vortexed. The mixture was subsequently stirred at 80 °C for 2 hours to generate the prepolymer. The vial was returned to the glovebox, and the prepolymer was diluted with 12 mL of dry THF. Separately, 1,6-hexanediol, glycerol, **$Et_2Si(OC_6H_{12}OH)_2$**, and **$EtSi(OC_6H_{12}OH)_3$** were weighed into individual 4-mL vials (see below for amounts and stoichiometries). The components were dissolved and combined in a total of 3 mL of dry THF, and the resulting solution was transferred into a 6-mL syringe. While vortexing, 8 mL of dry chloroform was added to the 40 mL vial containing the prepolymer, followed by the 3 mL solution of 1,6-hexanediol, glycerol, **$Et_2Si(OC_6H_{12}OH)_2$**, and **$EtSi(OC_6H_{12}OH)_3$**. An additional 1 mL of dry THF was used to rinse the syringe to ensure complete transfer. The resulting mixture was vortexed for approximately 20 seconds, after which it was poured into a Teflon mold. The solvent was allowed to evaporate slowly in the glovebox for 24 hours, forming a uniform polyurethane film. The film was then further dried overnight in a 100 °C vacuum oven.

Table S3. Stoichiometries and Amounts of Reagents for BCS and TCJ-based PU Networks

	1,6-Hexanediol (mg / mmol / equiv)	$Et_2Si(OC_6H_{12}OH)_2$ (mg / mmol / equiv)	Glycerol (mg / mmol / equiv)	$EtSi(OC_6H_{12}OH)_3$ (mg / mmol / equiv)
**vPU**	175.5 / 1.485 / 0.55	–	74.6 / 0.81 / 0.30	–
**dPU-BCS$_{0.4}$**	31.9 / 0.27 / 0.10	389.5 / 1.215 / 0.45	74.6 / 0.81 / 0.30	–
**dPU-BCS$_{0.3}$TCJ$_{0.05}$**	79.8 / 0.675 / 0.25	259.6 / 0.81 / 0.30	62.2 / 0.675 / 0.25	55.2 / 0.135 / 0.05
**dPU-BCS$_{0.2}$TCJ$_{0.083}$**	111.7 / 0.945 / 0.35	173.1 / 0.54 / 0.20	53.9 / 0.585 / 0.217	91.9 / 0.225 / 0.083
**dPU-BCS$_{0.1}$TCJ$_{0.117}$**	143.6 / 1.215 / 0.45	86.5 / 0.27 / 0.10	45.6 / 0.495 / 0.183	128.7 / 0.315 / 0.117
**dPU-TCJ$_{0.15}$**	175.5 / 1.485 / 0.55	–	37.3 / 0.405 / 0.15	165.5 / 0.405 / 0.15

3.4. Network Deconstruction

3.4.1. Small scale reactions for % insoluble mass measurement

A stock solution of $FeCl_3$ (50 mM) in MeOH was prepared. To each scintillation vial containing a small stir bar and the dry network after the gel fraction measurement experiments was added 9 mL of THF and 1 mL of the $FeCl_3$ solution. The reactions were allowed to stir at 50 ºC for 12 h. The solution was removed and replaced with ~20 mL of fresh THF. This process was repeated two times for every 12 h. After 2 cycles of solvent replacement, the solvent was removed and the vials were dried at 100 °C for 24 h. The percent of mass recovery after network deconstruction experiment was calculated as:

$$\text{Insoluble mass (\%)} = \frac{\text{Mass of dry sample}}{\text{Mass of original sample}} \times 100\%$$

3.4.2. Large scale reactions for recycling experiments

PU networks (6.33 g) were cut into small pieces and added to a 1000-mL round bottom flask containing a stir bar. 500 mL solution of $FeCl_3$ (5 mM) in THF/MeOH (9/1, v/v) was added, and the reaction mixture was allowed to stir at 50 ºC for 24 h until no residual solid was observed. The mixture was then concentrated under reduced pressure to remove the solvent, and the residue was redissolved in chloroform. This solution was washed extensively with water (at least 10 times) to remove $FeCl_3$ and colored impurities. The organic layer was dried over magnesium sulfate for 1 h, and the collected solution was concentrated under reduced pressure and dried at 100 °C for 24 h to obtain deconstructed fragments (5.1 g, 81% recovery yield).

Similar procedure was used for the deconstruction reaction of 1st-gen recycled network. The recovery yield of the fragments was 85%.

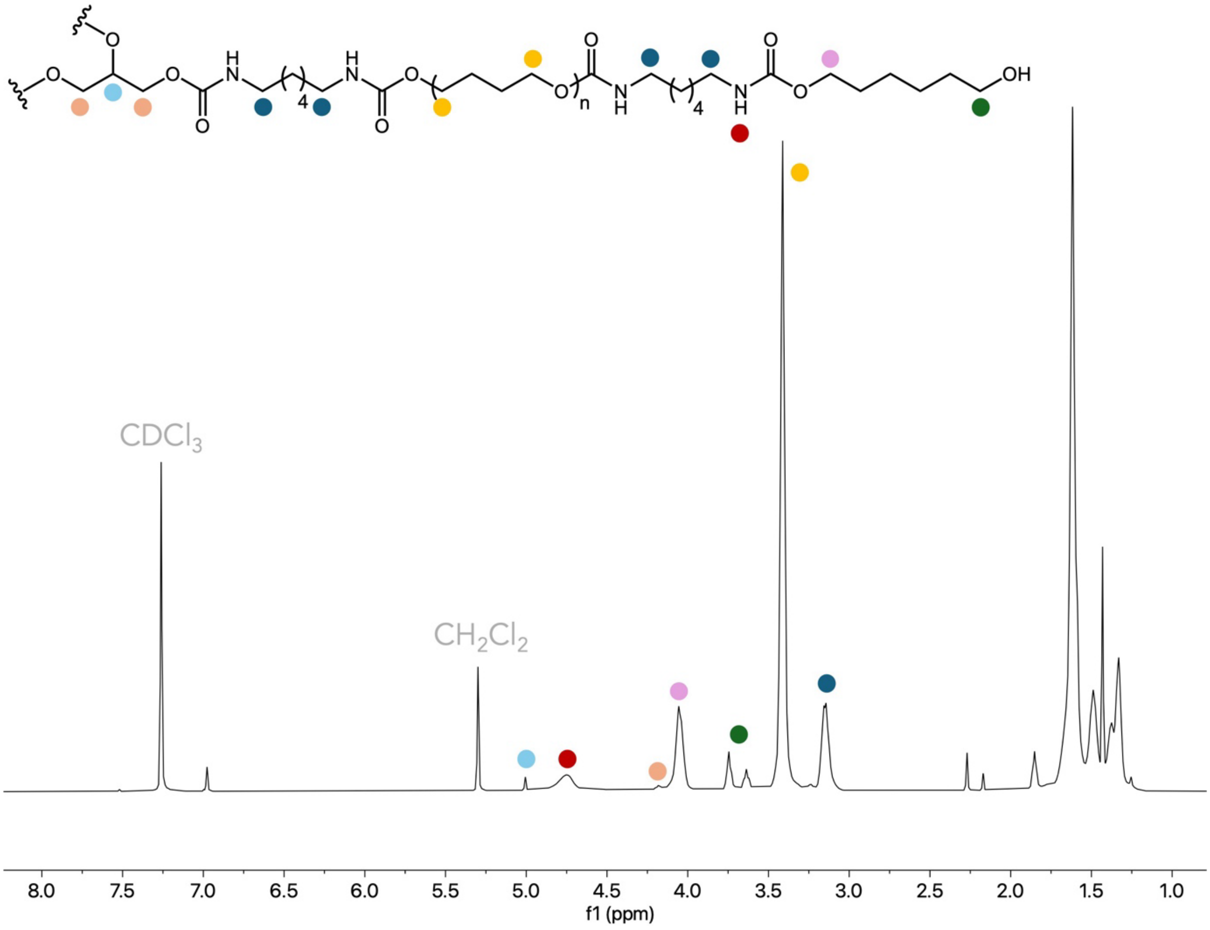

Figure S3. NMR spectrum of deconstructed fragments after purification via aqueous workup.

3.4.3. Quantification of –OH content

To a 1-dram vial was added ~20–30 mg deconstructed fragments (exact mass recorded) and a small stir bar. Deuterated chloroform (~0.5 mL) and an internal standard (mesitylene, 0.025 mmol, 3.5 μL) were added, and the mixture was vortexed until the fragments were fully dissolved. Pyridine (30 μmol, 3 μL) and trifluoroacetic anhydride (30 μmol, 5 μL) were added. The amounts of pyridine and trifluoroacetic anhydride correspond to ~3.0 equiv of the theoretical –OH content based on the amount of cleavable additive used for the network synthesis. The reaction was stirred at ambient temperature for 2 h before being subjected to NMR analysis. The ratio of the internal standard to the $–CH_2–$ group at the α position of the trifluoroacetyl group was used to calculate the –OH content per unit mass of the fragments. This measurement was performed on 3 samples to determine the average –OH content in the fragments.

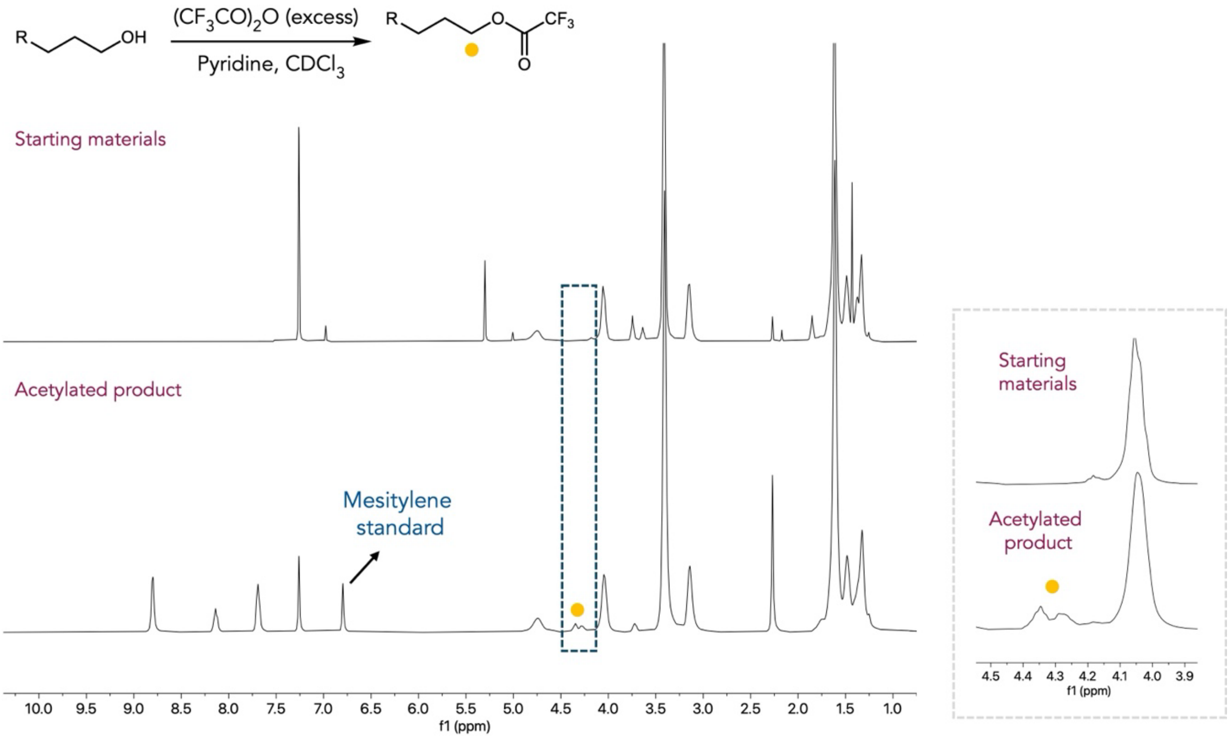

Figure S4. ^{1}H NMR spectra showing the acetylation reaction of the fragments for –OH quantification.

3.5. Chemical Recycling

3.5.1. Synthesis of **rPU-TCJ-G0**

1.755 g of polyTHF (MW = 650, 2.7 mmol, 1.0 equiv) and 908 mg of HMDI (5.4 mmol, 2.0 equiv) were added to a 40-mL vial containing a magnetic stir bar inside a glovebox, and the mixture was stirred thoroughly. The vial was then sealed with a penetrable cap and electrical tape and removed from the glovebox. Using a syringe, 80 μL of DBTDL (0.135 mmol, 0.05 equiv) was added, and the mixture was vortexed. The mixture was subsequently stirred at 80 °C for 2 hours to generate the prepolymer. The vial was returned to the glovebox, and the prepolymer was diluted with 12 mL of dry THF. Separately, 175.5 mg 1,6-hexanediol (1.485 mmol, 0.55 equiv), 24.9 mg glycerol (0.27 mmol, 0.10 equiv), and 220.7 mg **$EtSi(OC_6H_{12}OH)_3$** (0.54 mmol, 0.20 equiv) were weighed into individual 4-mL vials. The components were dissolved and combined in a total of 3 mL of dry THF, and the resulting solution was transferred into a 6-mL syringe. While vortexing, 8 mL of dry chloroform was added to the 40 mL vial containing the prepolymer, followed by the 3 mL solution of 1,6-hexanediol, glycerol, and **$EtSi(OC_6H_{12}OH)_3$**. An additional 1 mL of dry THF was used to rinse the syringe to ensure complete transfer. The resulting mixture was vortexed for approximately 20 seconds, after which it was poured into a Teflon mold. The solvent was allowed to evaporate slowly in the glovebox for 24 hours, forming a uniform polyurethane film. The film was then further dried overnight in a 100 °C vacuum oven.

3.5.2. Synthesis of **rPU-TCJ-G1(38)**

1.17 g of polyTHF (MW = 650, 1.80 mmol, 1.0 equiv) and 605.5 mg of HMDI (3.60 mmol, 2.0 equiv) were added to a 40-mL vial containing a magnetic stir bar inside a glovebox, and the mixture was stirred thoroughly. The vial was then sealed with a penetrable cap and electrical tape before being removed from the glovebox. Using a syringe, 53 μL of DBTDL (0.089 mmol, 0.05 equiv) was added, and the mixture was vortexed. The reaction mixture was subsequently stirred at 80 °C for 2 hours to generate the prepolymer. The vial was returned to the glovebox, and the prepolymer was diluted with 12 mL of dry THF. Separately, 76 mg of 1,6-hexanediol (0.643 mmol, 0.36 equiv) and 217.9 mg **$EtSi(OC_6H_{12}OH)_3$** (0.533 mmol, 0.30 equiv) were weighed into individual 4-mL vials. The components were dissolved and combined in a total of 3 mL of dry THF, and the resulting solution was transferred into a 3 mL syringe. Additionally, 1.288 g of **dPU-TCJ-G0** fragments (0.555 mmol/g hydroxyl group) was weighed into a separate 20-mL vial, dissolved in 7 mL of dry chloroform, and the resulting solution was transferred into a 12-mL syringe. While vortexing, the fragment solution was added to the 40-mL vial containing the prepolymer. An additional 1 mL of dry chloroform was used to rinse the 12-mL syringe to ensure complete transfer. Then, a 3-mL solution of 1,6-hexandiol and **$EtSi(OC_6H_{12}OH)_3$** was added to the 40-mL vial under vigorous vortexing, and an additional 1 mL of dry THF was used to rinse the 3-mL syringe. The resulting mixture was vortexed for approximately 20 seconds before being poured into a Teflon mold. The solvent was allowed to evaporate slowly in the glovebox for 24 hours, forming a uniform polyurethane film, which was further dried overnight in a 100 °C vacuum oven.

3.5.3. Synthesis of **rPU-TCJ-G2(23)**

1.443 g of polyTHF (MW = 650, 2.22 mmol, 1.0 equiv) and 747.3 mg of HMDI (4.44 mmol, 2.0 equiv) were added to a 40-mL vial containing a magnetic stir bar inside a glovebox, and the mixture was stirred thoroughly. The vial was then sealed with a penetrable cap and electrical tape before being removed from the glovebox. Using a syringe, 66 μL of DBTDL (0.111 mmol, 0.05 equiv) was added, and the mixture was vortexed. The reaction mixture was subsequently stirred at 80 °C for 2 hours to generate the prepolymer. The vial was returned to the glovebox, and the prepolymer was diluted with 12 mL of dry THF. Separately, 93.8 mg of 1,6-hexanediol (0.784 mmol, 0.36 equiv) and 268.9 mg **$EtSi(OC_6H_{12}OH)_3$** (0.658 mmol, 0.30 equiv) were weighed into individual 4-mL vials. The components were dissolved and combined in a total of 3 mL of dry THF, and the resulting solution was transferred into a 3 mL syringe. Additionally, 780 mg of **rPU-TCJ-G1(38)** fragments (1.13 mmol/g hydroxyl group) was weighed into a separate 20-mL vial, dissolved in 7 mL of dry chloroform, and the resulting solution was transferred into a 12-mL syringe. While vortexing, the fragment solution was added to the 40-mL vial containing the prepolymer. An additional 1 mL of dry chloroform was used to rinse the 12-mL syringe to ensure complete transfer. Then, a 3-mL solution of 1,6-hexandiol and **$EtSi(OC_6H_{12}OH)_3$** was added to the 40-mL vial under vigorous vortexing, and an additional 1 mL of dry THF was used to rinse the 3-mL syringe. The resulting mixture was vortexed for approximately 20 seconds before being poured into a Teflon mold. The solvent was allowed to evaporate slowly in the glovebox for 24 hours, forming a uniform polyurethane film, which was further dried overnight in a 100 °C vacuum oven.

3.5.4. Synthesis of **rPU-TCJ-G2(47)**

1.042 g of polyTHF (MW = 650, 1.603 mmol, 1.0 equiv) and 539.4 mg of HMDI (3.206 mmol, 2.0 equiv) were added to a 40-mL vial containing a magnetic stir bar inside a glovebox, and the mixture was stirred thoroughly. The vial was then sealed with a penetrable cap and electrical tape before being removed from the glovebox. Using a syringe, 48 μL of DBTDL (0.081 mmol, 0.05 equiv) was added, and the mixture was vortexed. The reaction mixture was subsequently stirred at 80 °C for 2 hours to generate the prepolymer. The vial was returned to the glovebox, and the prepolymer was diluted with 12 mL of dry THF. Separately, 194.1 mg of **$EtSi(OC_6H_{12}OH)_3$** (0.475 mmol, 0.30 equiv) was weighed into a 4-mL vial, dissolved in 3 mL of dry THF, and the resulting solution was transferred into a 3-mL syringe. Additionally, 1.577 g of **rPU-TCJ-G1(38)** fragments (1.13 mmol/g hydroxyl group) was weighed into a separate 20-mL vial, dissolved in 7 mL of dry chloroform, and the resulting solution was transferred into a 12-mL syringe. While vortexing, the fragment solution was added to the 40-mL vial containing the prepolymer. An additional 1 mL of dry chloroform was used to rinse the 12-mL syringe to ensure complete transfer. Then, a 3-mL solution of **$EtSi(OC_6H_{12}OH)_3$** was added to the 40-mL vial under vigorous vortexing, and an additional 1 mL of dry THF was used to rinse the 3-mL syringe. The resulting mixture was vortexed for approximately 20 seconds before being poured into a Teflon mold. The solvent was allowed to evaporate slowly in the glovebox for 24 hours, forming a uniform polyurethane film, which was further dried overnight in a 100 °C vacuum oven.

4. Supplementary Data

4.1. FTIR

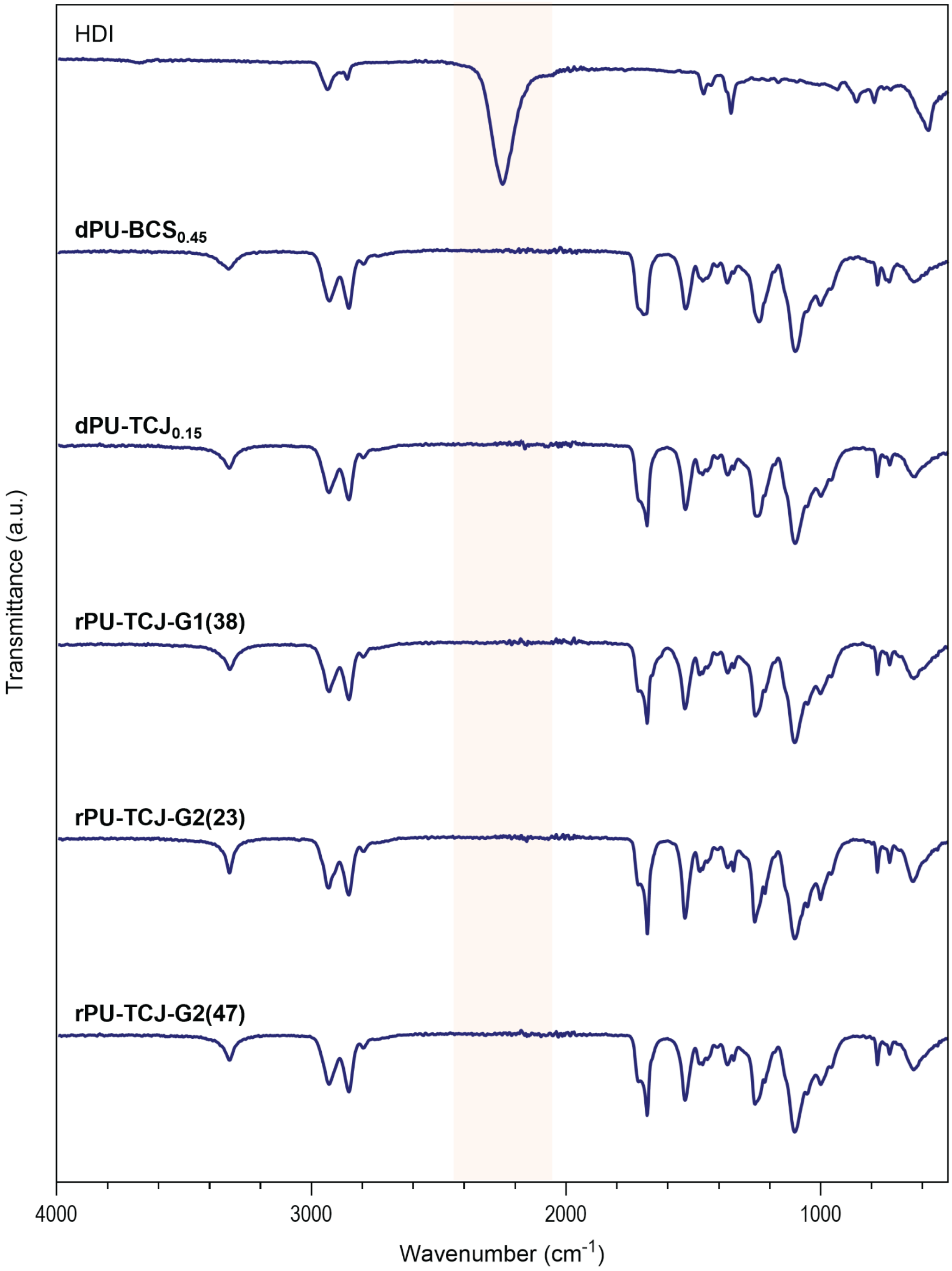

Figure S5. FTIR spectra of HDI and polyurethane networks, with the isocyanate peak highlighted to indicate complete conversion within the networks.

4.2. Tensile Testing

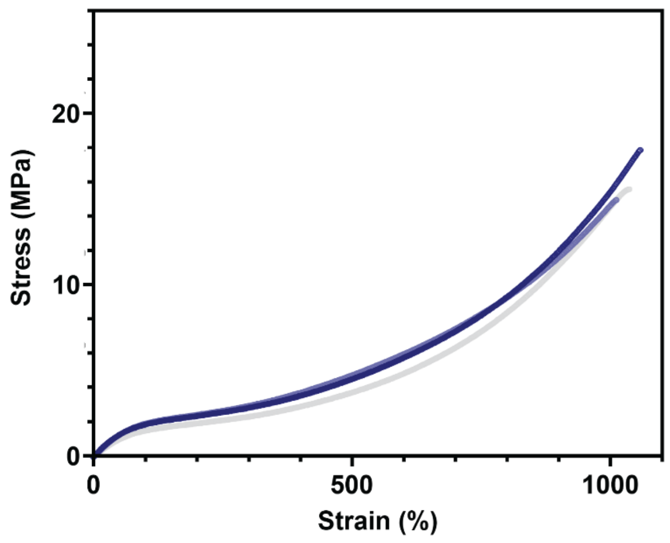

Figure S6. Uniaxial tensile stress-strain curves for **vPU**.

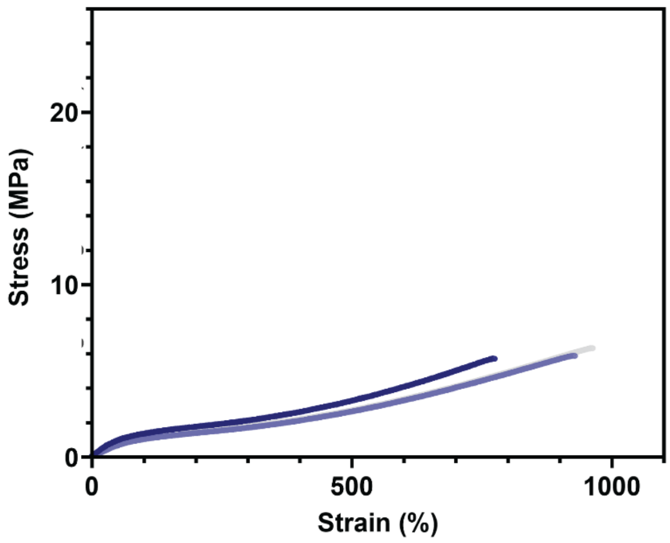

Figure S7. Uniaxial tensile stress-strain curves for **dPU-BCS$_{0.45}$**.

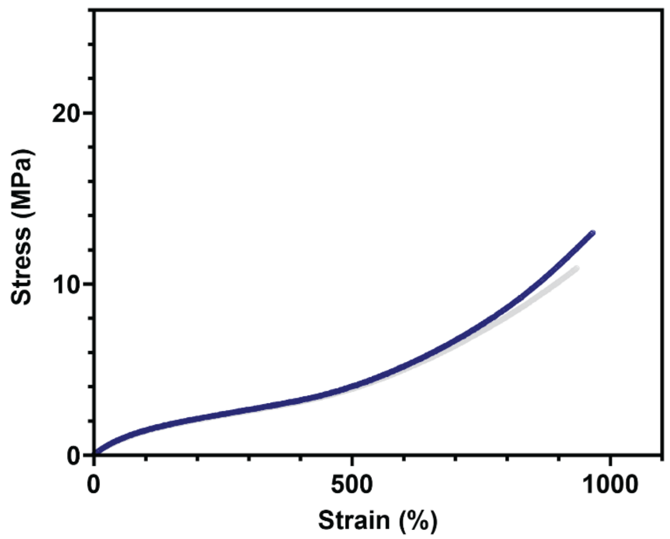

Figure S8. Uniaxial tensile stress-strain curves for **dPU-BCS$_{0.3}$TCJ$_{0.05}$**.

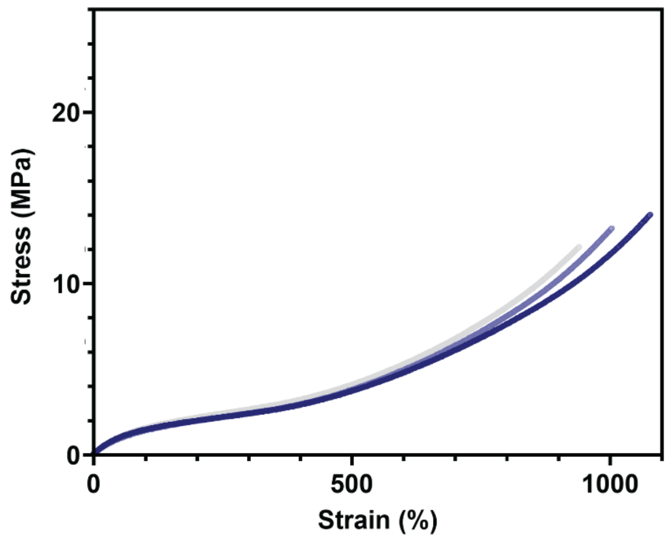

Figure S9. Uniaxial tensile stress-strain curves for **dPU-BCS$_{0.2}$TCJ$_{0.083}$**.

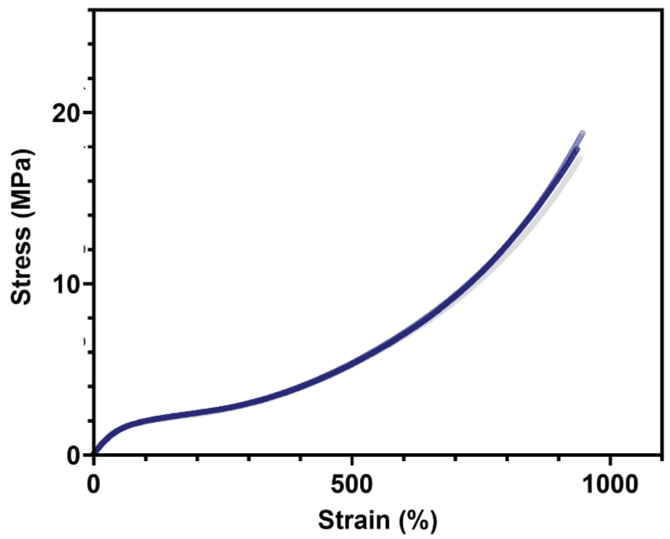

Figure S10. Uniaxial tensile stress-strain curves for **dPU-BCS$_{0.1}$TCJ$_{0.117}$**.

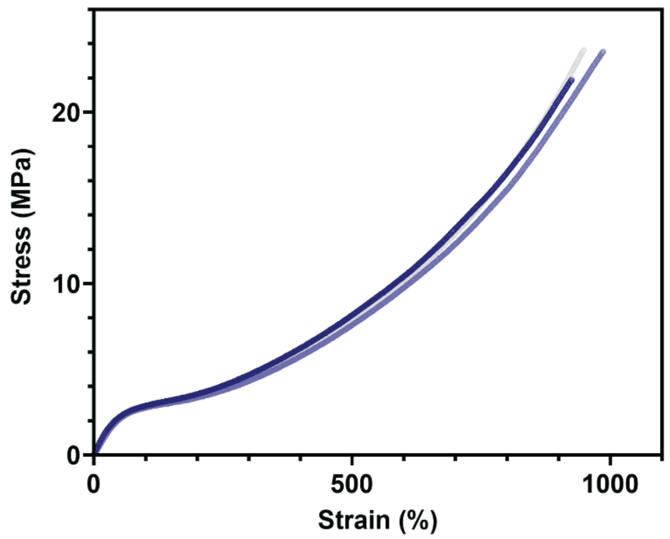

Figure S11. Uniaxial tensile stress-strain curves for **dPU-TCJ$_{0.15}$**.

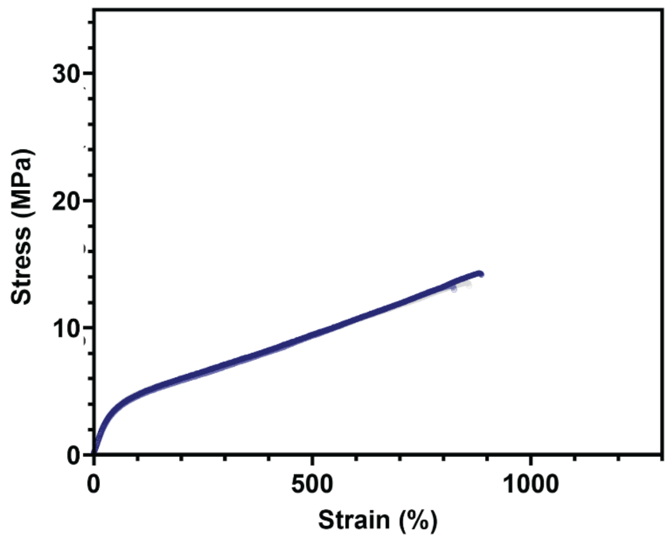

Figure S12. Uniaxial tensile stress-strain curves for **dPU-TCJ-G0**.

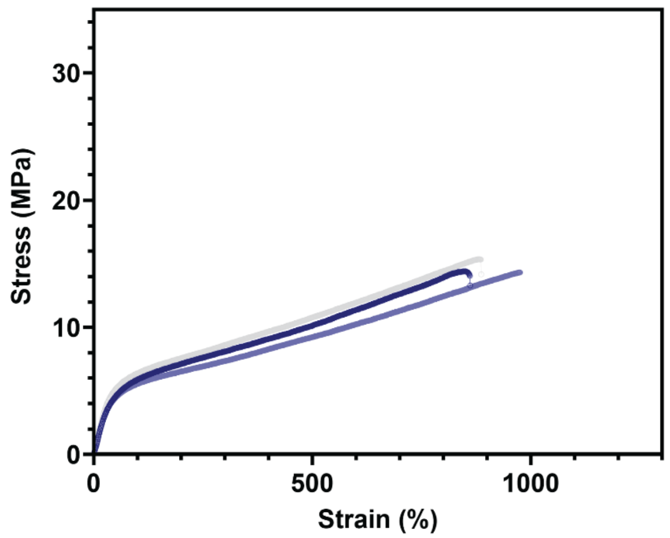

Figure S13. Uniaxial tensile stress-strain curves for **rPU-TCJ-G1(38)**.

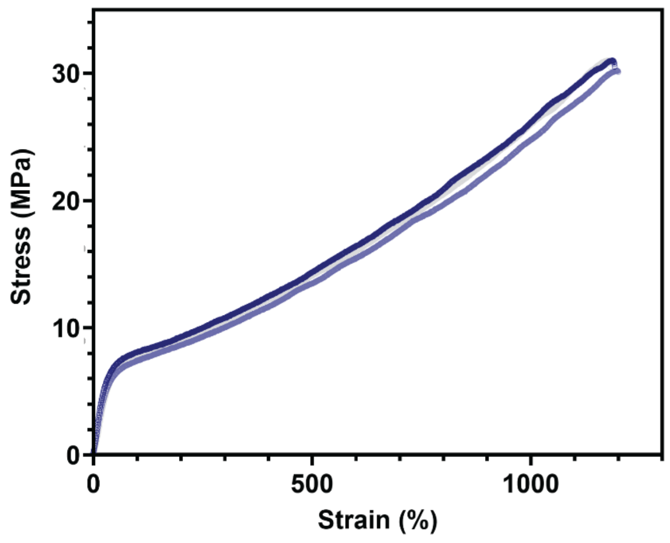

Figure S14. Uniaxial tensile stress-strain curves for **rPU-TCJ-G2(23)**.

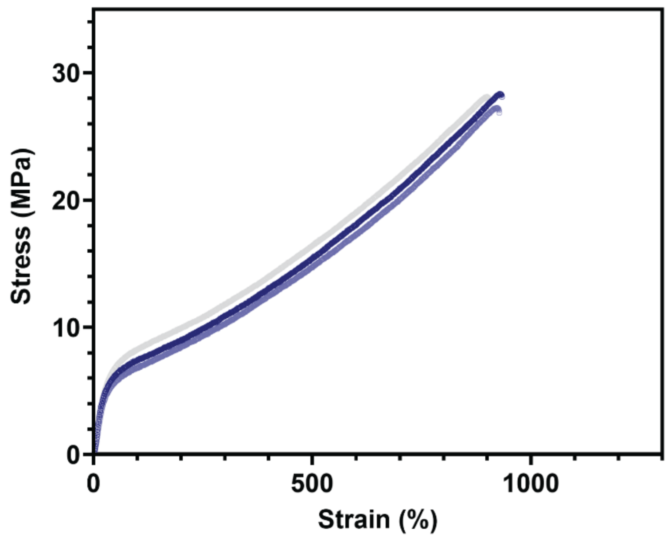

Figure S15. Uniaxial tensile stress-strain curves for **rPU-TCJ-G2(47)**.

4.3. DMA

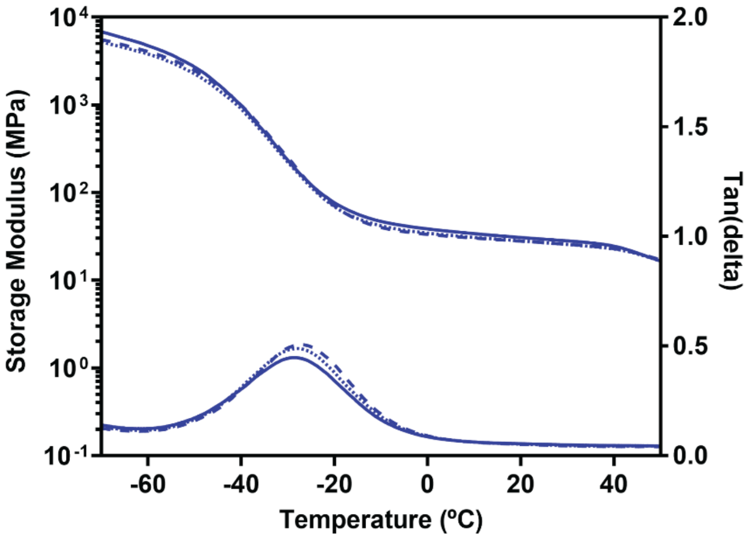

Figure S16. DMA temperature sweep at constant amplitude and frequency for **vPU**.

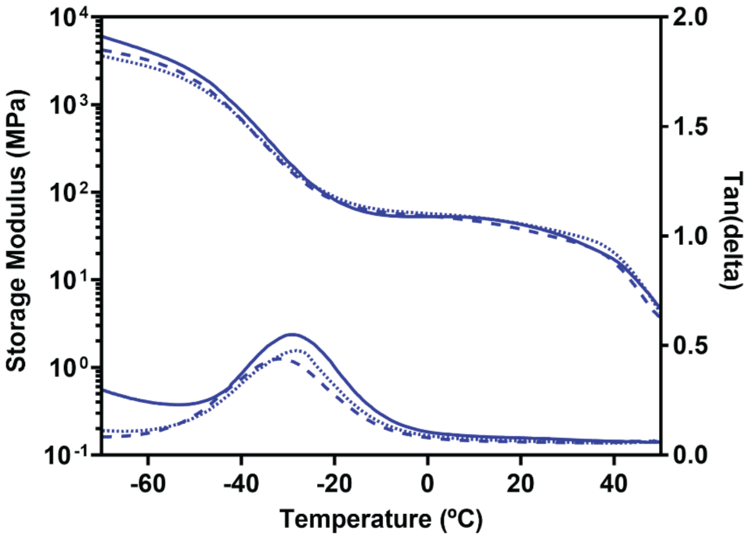

Figure S17. DMA temperature sweep at constant amplitude and frequency for **dPU-BCS$_{0.45}$**.

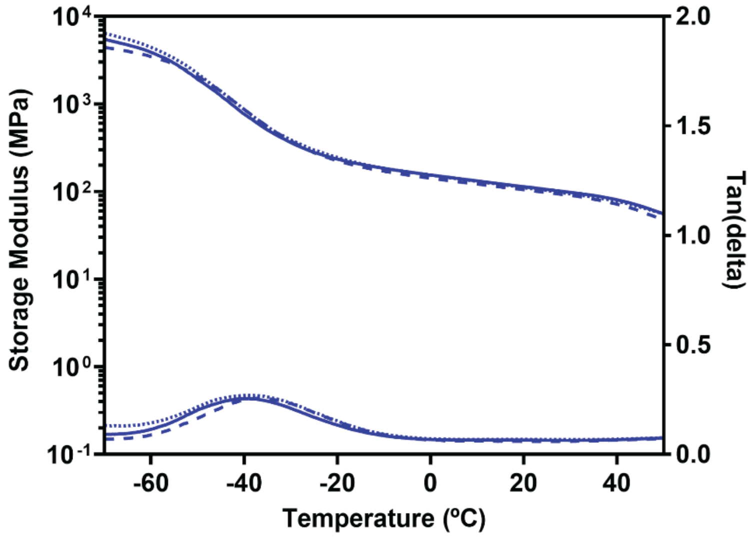

Figure S18. DMA temperature sweep at constant amplitude and frequency for **dPU-TCJ$_{0.15}$**.

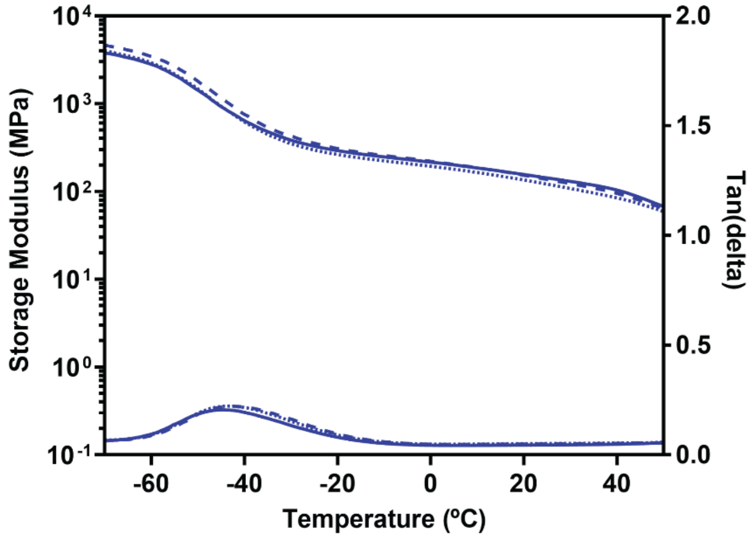

Figure S19. DMA temperature sweep at constant amplitude and frequency for **rPU-TCJ-G1(38)**.

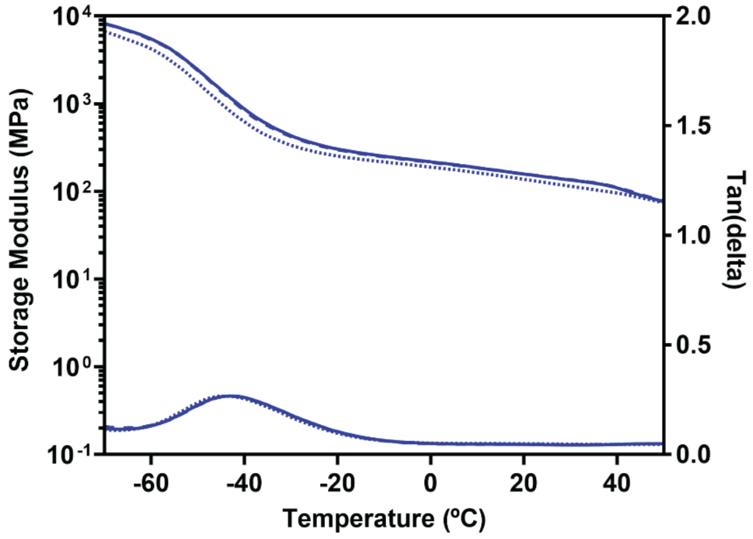

Figure S20. DMA temperature sweep at constant amplitude and frequency for **rPU-TCJ-G2(23)**.

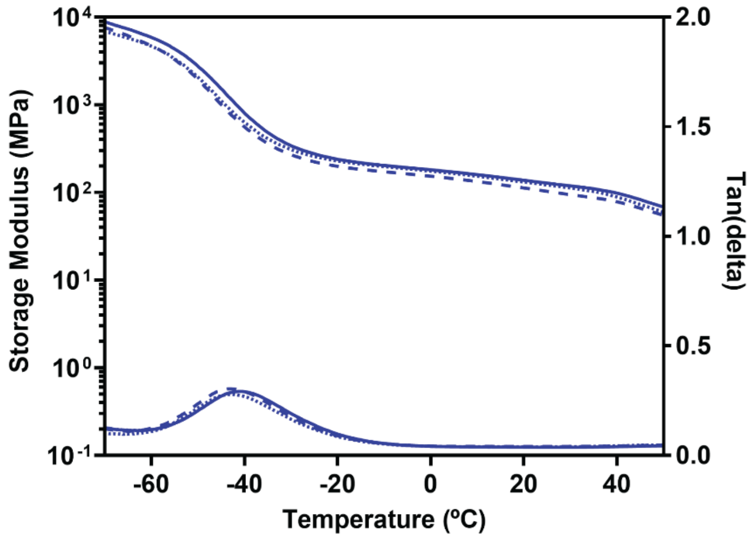

Figure S21. DMA temperature sweep at constant amplitude and frequency for **rPU-TCJ-G2(47)**.

4.4. TGA

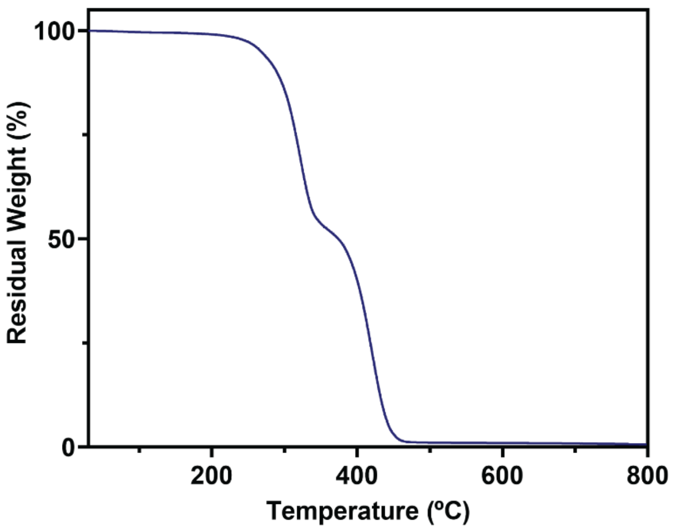

Figure S22. Thermal stability of **vPU** analyzed by TGA.

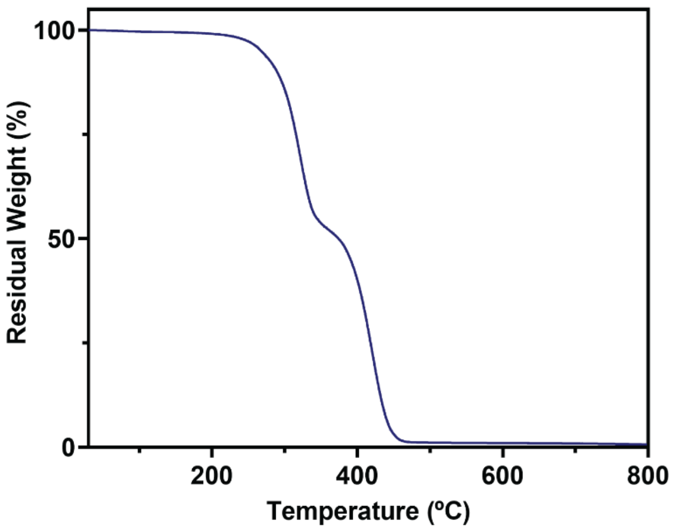

Figure S23. Thermal stability of **dPU-BCS$_{0.45}$** analyzed by TGA.

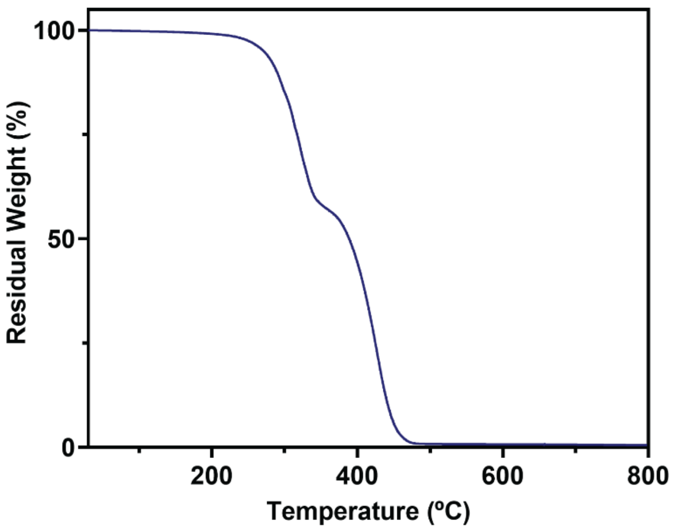

Figure S24. Thermal stability of **dPU-TCJ$_{0.3}$** analyzed by TGA.

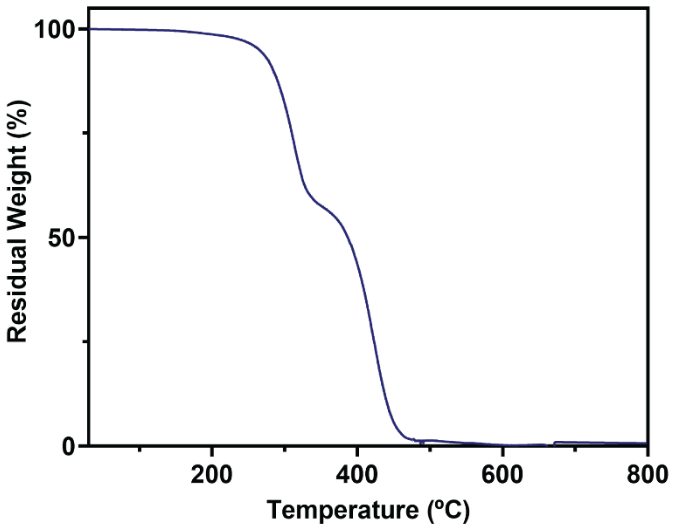

Figure S25. Thermal stability of **rPU-TCJ-G1(38)** analyzed by TGA.

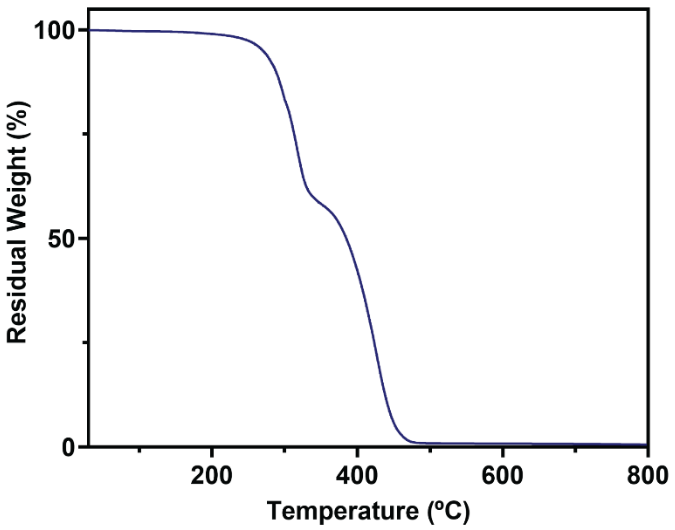

Figure S26. Thermal stability of **rPU-TCJ-G2(23)** analyzed by TGA.

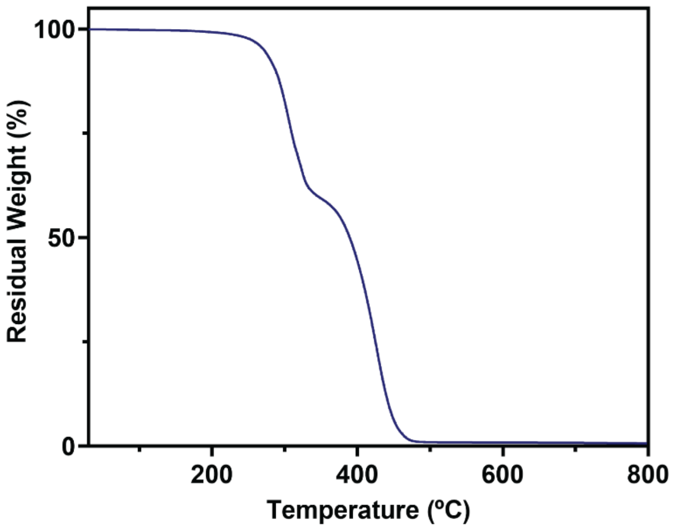

Figure S27. Thermal stability of **rPU-TCJ-G2(47)** analyzed by TGA.

4.5. Deconstruction Experiments

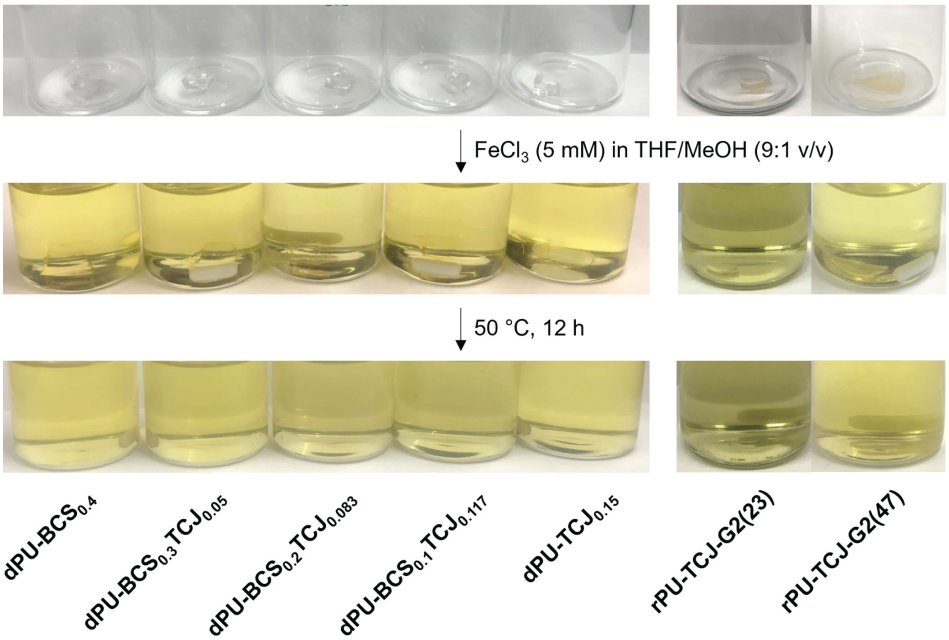

Figure S28. $FeCl_3$-mediated deconstruction of polyurethane networks. Complete dissolution was observed for all materials.

4.6. SEC

4.6.1. Experimental measurement of fragment molar mass

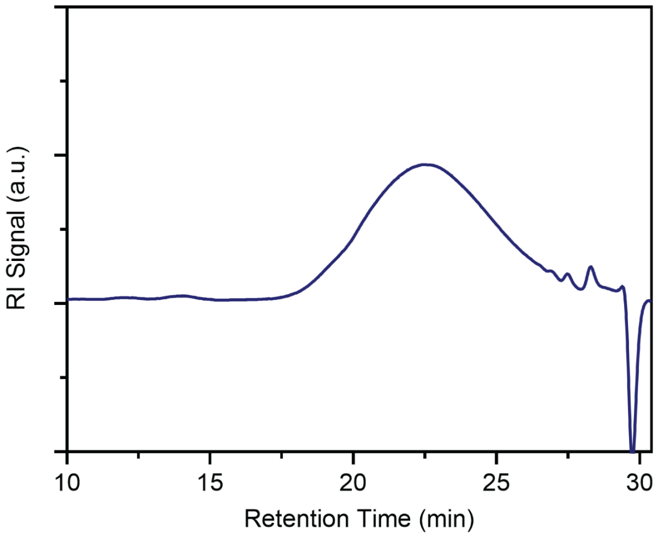

Figure S29. SEC analysis of **dPU-TCJ-G0** fragments. Molar masses were referenced against polystyrene standards (M_n = 5.6 kDa, M_w = 27.0 kDa, $Đ$ = 4.8)

4.6.2. Theoretical determination of fragment molar mass

dPU-TCJ-G0 consists of 1.0 equivalent of prepolymer, 0.55 equivalents of 1,6-hexanediol, 0.1 equivalents of glycerol, and 0.2 equivalents of **$EtSi(OC_6H_{12}OH)_3$**.

Following complete cleavage of all Si–O bonds, the system comprises 1.0 equivalent of B_2, 0.55 equivalents of A_2, 0.1 equivalents of A_3, and 0.6 equivalents of A. Here, A represents a hydroxyl group, B denotes an isocyanate group, and the subscript indicates the functionality of each fragment component.

By analyzing the connectivity of reactive groups within these fragment components, the average molecular weight and effective functionality of the resulting fragments can be theoretically determined.

The total number of fragment components prior to bond formation is:

1.0 + 0.55 + 0.1 + 0.6 = 2.25 equivalents

Within the system, the total number of A groups is:

1.1 (from A_2 and A_3) + 0.6 (from A) + 0.3 (from A_3) = 2.0 equivalents,

which is the same as the total number of B groups.

Each bond formed results from the reaction between one A group and one B group. Therefore, the total number of bonds formed is equal to the number of reactive A (or B) groups, which is 2.0 equivalents.

In step-growth polymerization without ring closure, each bond reduces the total number of fragments (or "clusters") by one. Hence, the number of fragments at full conversion is:

Number of fragments = Number of initial fragment components − Number of bonds formed = 2.25 − 2.0 = 0.25 equivalents

Therefore, on average, each fragment contains:

1.0 / 0.25 = 4.0 prepolymer units

0.55 / 0.25 = 2.2 hexanediol units

0.1 / 0.25 = 0.4 glycerol units

0.6 / 0.25 = 2.4 hexanediol units originating from the cleavage of **$EtSi(OC_6H_{12}OH)_3$**

Each prepolymer unit contains, on average, one pTHF and two HDI units. Plugging in the molar masses of the fragment components yields a number-average molar mass of ~4.5 kDa for the resulting fragments. This value agrees well with the experimentally measured 5.6 kDa; the small disparity may be due to inaccuracies when referencing molar masses to polystyrene standards or the minimal loss of small-molar-mass fragments during workup.

The hydroxyl termini of the fragments originate exclusively from the cleavage of **$EtSi(OC_6H_{12}OH)_3$**. Accordingly, the effective functionality of the fragments is 2.4, corresponding to an average of 2.4 hydroxyl groups per fragment.

4.6.3. Fragment molar mass after recycling

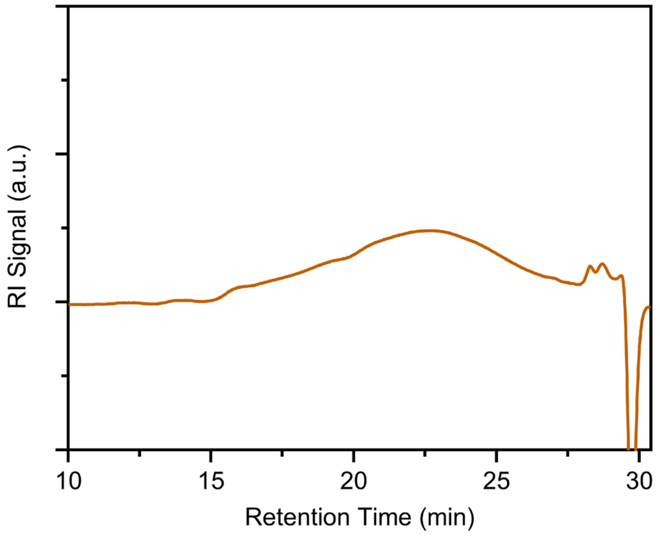

Figure S30. SEC analysis of **dPU-TCJ-G1** fragments. Molar masses were referenced against polystyrene standards (M_n = 4.5 kDa, M_w = 62.6 kDa, $Đ$ = 14.0). The increase in weight-average molar mass relative to the **dPU-TCJ-G0** fragment indicates the formation of larger species.

4.7. NMR

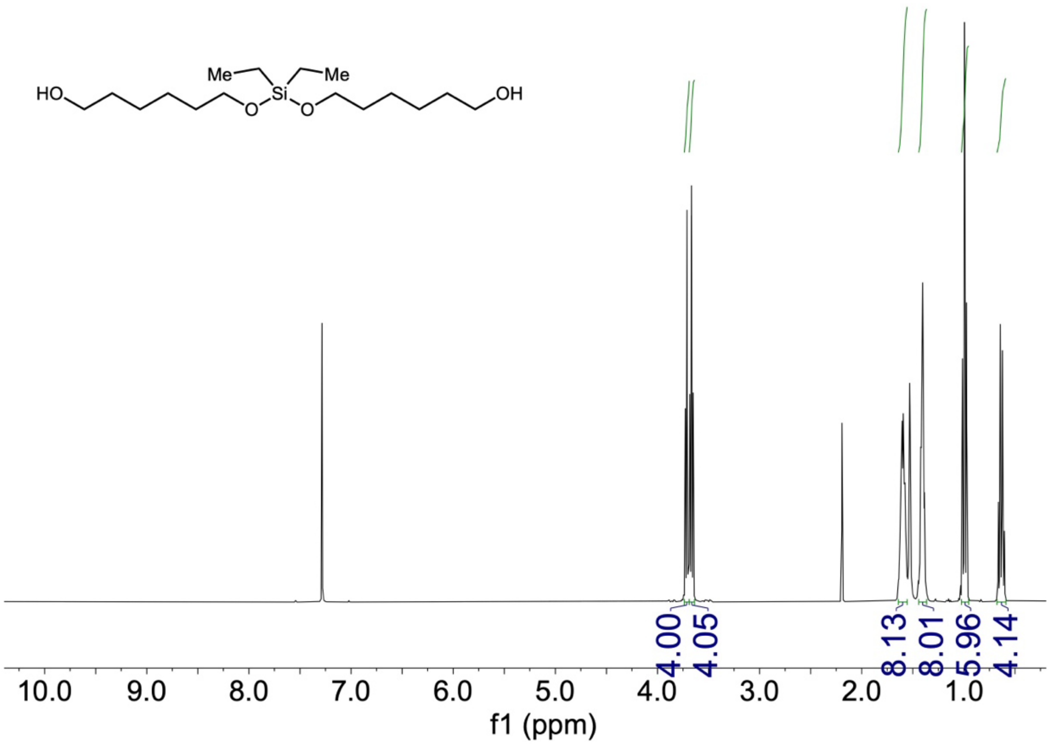

Figure S31. ^{1}H NMR spectrum of **$Et_2Si(OC_6H_{12}OH)_2$** ($CDCl_3$, 400 MHz, 25 °C).

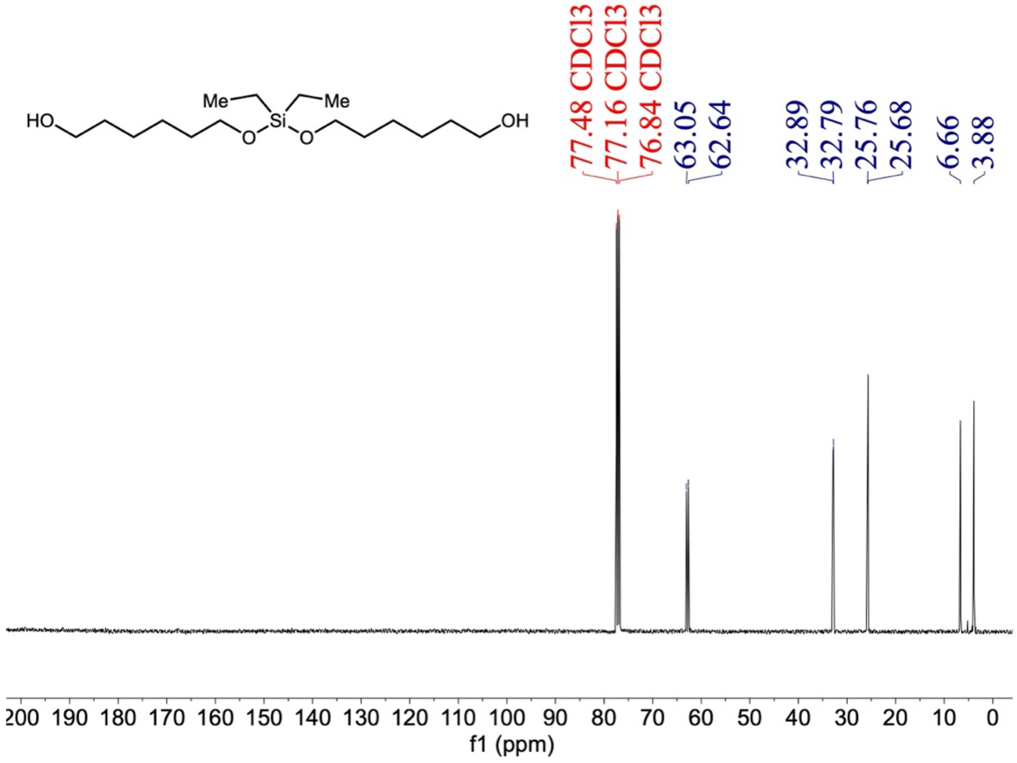

Figure S32. ^{13}C NMR spectrum of **$Et_2Si(OC_6H_{12}OH)_2$** ($CDCl_3$, 101 MHz, 25 °C).

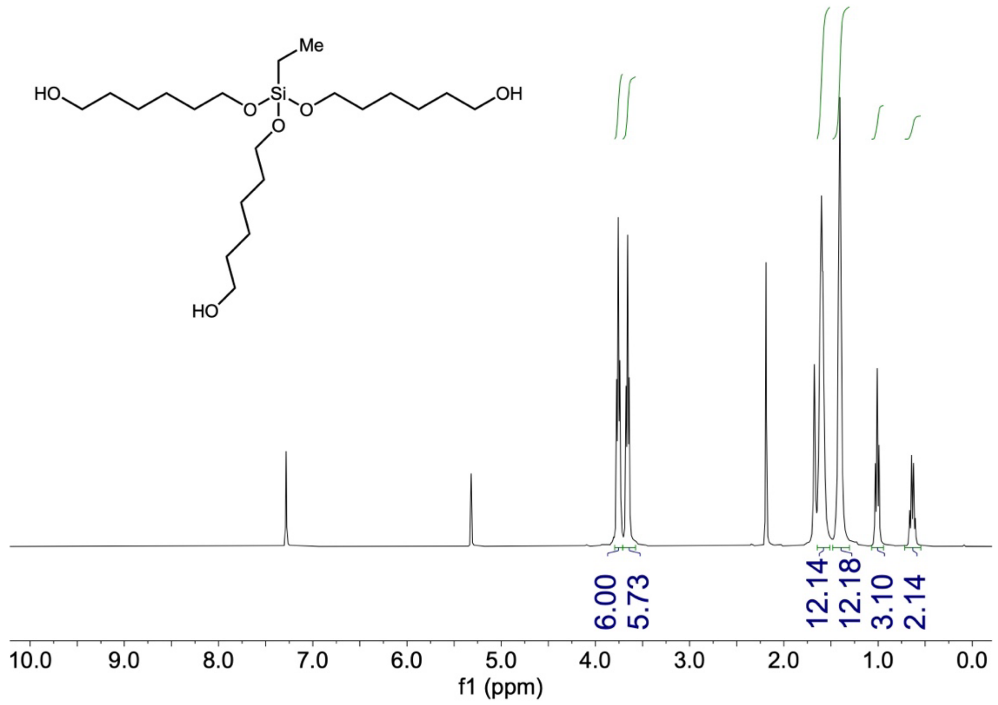

Figure S33. ^{1}H NMR spectrum of **EtSi(OC$_6$H$_{12}$OH)$_3$** (CDCl$_3$, 400 MHz, 25 °C).

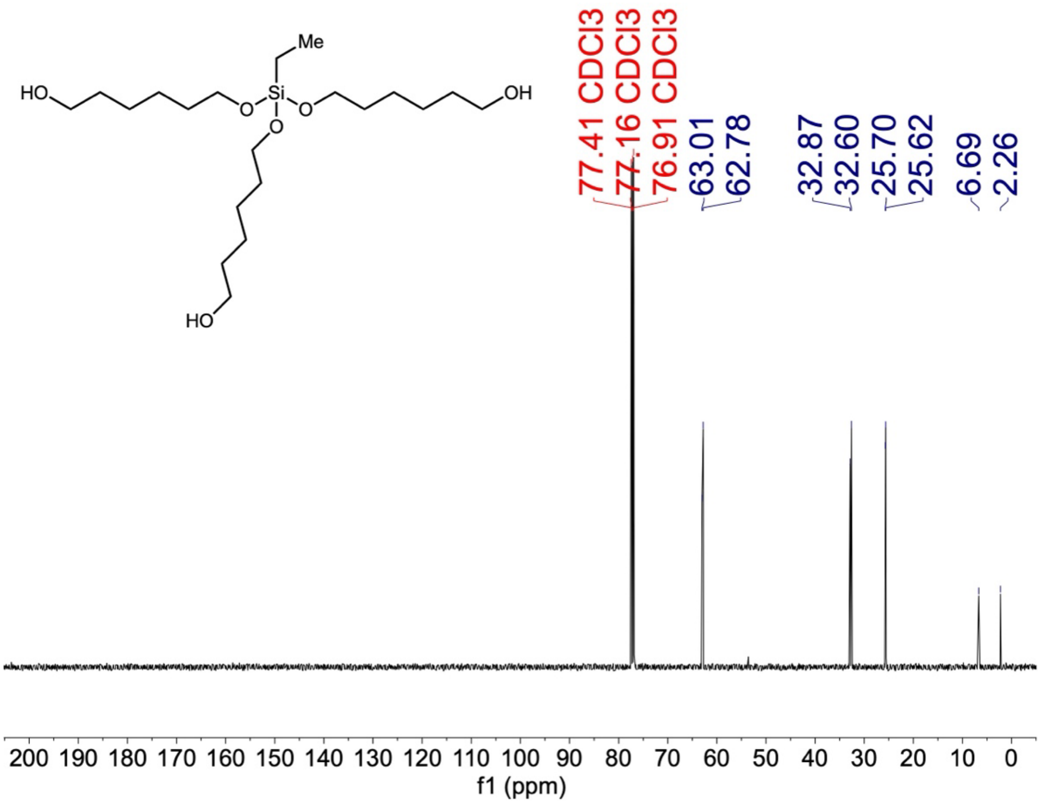

Figure S34. ^{13}C NMR spectrum of **EtSi(OC$_6$H$_{12}$OH)$_3$** (CDCl$_3$, 126 MHz, 25 °C).

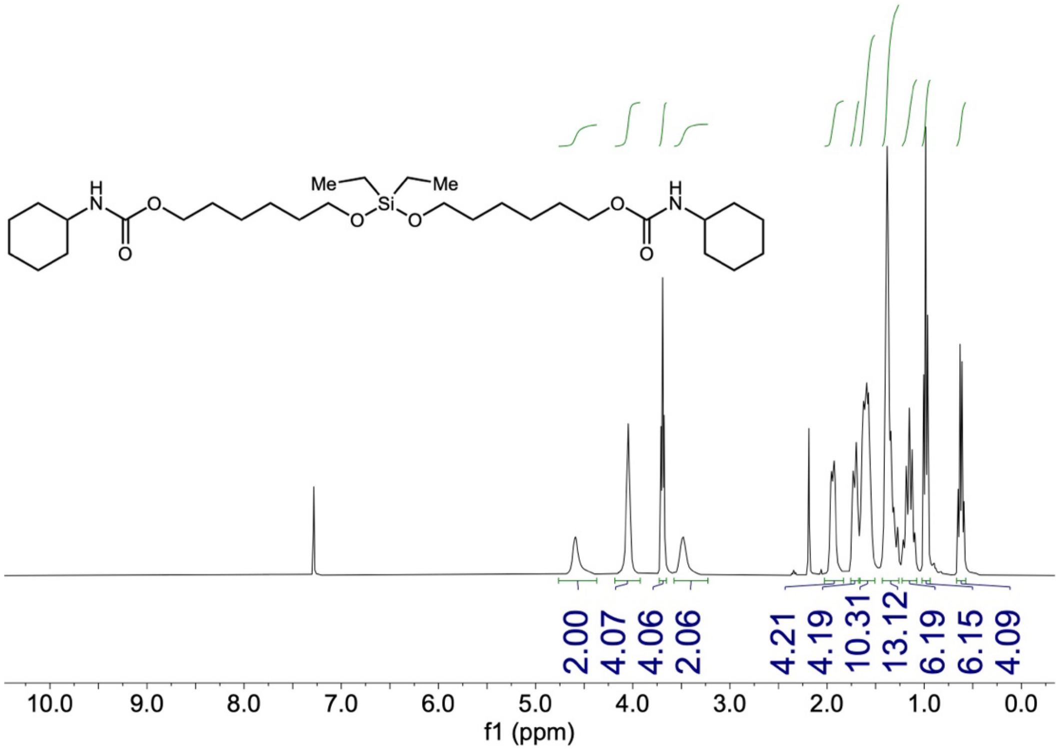

Figure S35. ^{1}H NMR spectrum of **$Et_2Si(OC_6H_{12}OC(O)NHCy)_2$** ($CDCl_3$, 400 MHz, 25 °C).

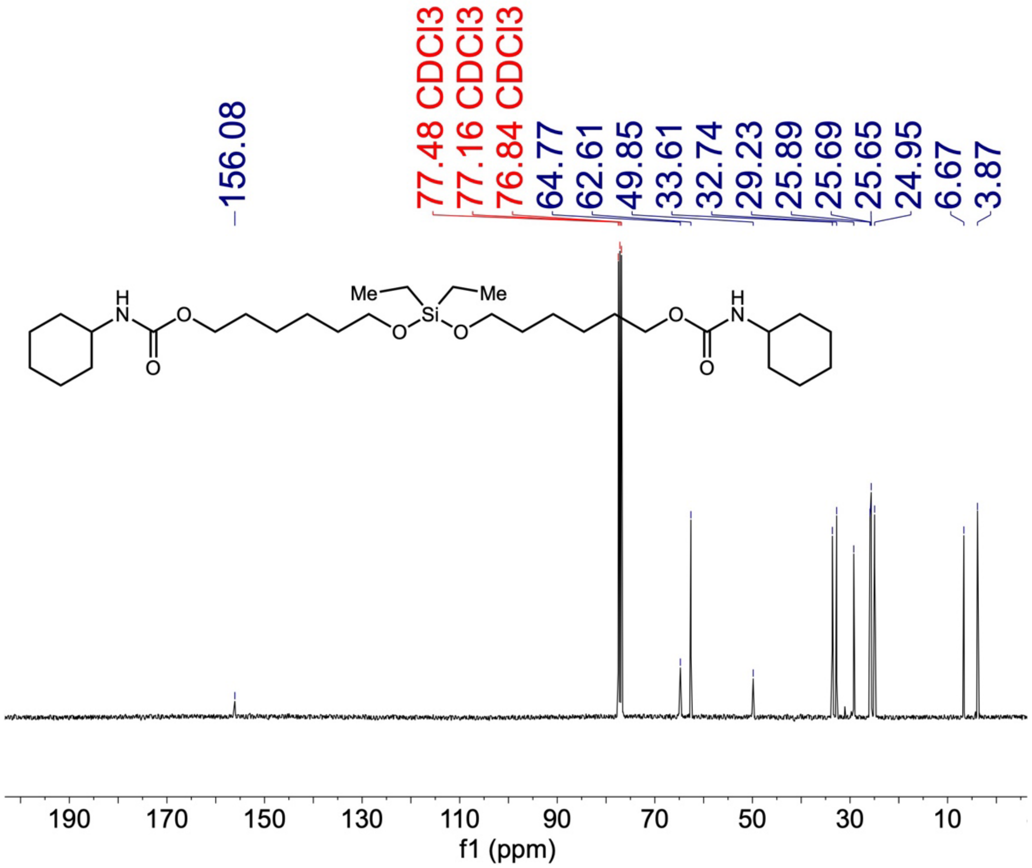

Figure S36. ^{13}C NMR spectrum of **$Et_2Si(OC_6H_{12}OC(O)NHCy)_2$** ($CDCl_3$, 101 MHz, 25 °C).

5. References

1. Macosko, C. W.; Miller, D. R. A New Derivation of Average Molecular Weights of Nonlinear Polymers. *Macromolecules* **1976**, *9*, 199–206.

2. Macosko, C. W.; Miller, D. R. A New Derivation of Post Gel Properties of Network Polymers. *Macromolecules* **1976**, *9*, 209–2011.
